# The association of obesity and lipid-related indicators with all-cause and cardiovascular mortality risks in patients with diabetes or prediabetes: a cross-sectional study based on machine learning algorithms

**DOI:** 10.3389/fendo.2025.1492082

**Published:** 2025-06-02

**Authors:** Zhaoqi Yan, Xing Chang, Zhiming Liu, Ruxiu Liu, Xiufan Du

**Affiliations:** ^1^ Guang'anmen Hospital, China Academy of Chinese Medical Sciences, Graduate School, Beijing, China; ^2^ The Third Hospital of Nanchang, Nanchang People's Hospital, Department of Rehabilitation Medicine, Nanchang, Jiangxi, China

**Keywords:** obesity and lipid-related indicators, triglyceride-glucose index, abdominal obesity index, visceral adiposity index, diabetes/prediabetes, national health and nutrition examination survey; machine learning algorithms

## Abstract

**Objective:**

This study aims to explore the associations between various obesity and lipid-related indicators in patients with diabetes or prediabetes. Specifically, the indicators examined include the triglyceride-glucose index (TyG), along with its derived metrics: TyG-BMI, TyG-WHtR, TyG-WWI, TyG-WC, lipid accumulation product (LAP), visceral adiposity index (VAI), and abdominal obesity index (ABSI), resulting in a total of eight indicators.

**Methods:**

This study utilizes data from the NHANES conducted from 1999 to 2018, analyzing a cohort of 4,058 patients diagnosed with diabetes/prediabetes. We utilized multivariable Cox regression models to evaluate the impact of these indicators on both all-cause and cardiovascular mortality rates. Additionally, we compared the predictive performance of eight machine learning (ML) algorithms regarding mortality risk and used the SHAP method to clarify the significance of obesity and lipid-related indicators in mortality prediction.

**Results:**

The results of the multivariable Cox regression analysis reveal significant associations between TyG, TyG-WWI, and ABSI with all-cause mortality among patients with diabetes/prediabetes. Compared to baseline levels, the HR for TyG in the fourth quartile (Q4) was 1.49, while for TyG-WWI (Q4), the HR was 1.52. Furthermore, ABSI was associated with increased all-cause mortality risk in groups Q3 and Q4, presenting risk ratios of 1.80 and 1.68, respectively. Notably, TyG (Q4) was also significantly associated with cardiovascular mortality risk, with an HR of 1.98. RCS analysis indicated a linear trend between TyG, TyG-WWI, and all-cause mortality, whereas ABSI displayed a non-linear trend. Among the ML algorithms evaluated, the XGBoost model exhibited the strongest predictive capability. The SHAP analysis indicated that the indicators with the greatest impact on all-cause mortality in patients with diabetes/prediabetes were ranked as follows: TyG > ABSI > TyG-WWI. Furthermore, sex-based subgroup analysis indicated that VAI was positively associated with cardiovascular mortality in male patients with diabetes/prediabetes, exhibiting a linear trend.

**Conclusion:**

TyG, TyG-WWI, ABSI, and VAI are closely linked to mortality risk in diabetes/prediabetes patients. Among these, TyG is significantly associated with both all-cause and cardiovascular mortality, showing superior predictive capability. We recommend long-term monitoring of these indicators and their inclusion in management strategies to effectively inform diabetes/prediabetes patients about their mortality risks.

## Introduction

The global prevalence of diabetes has reached alarming levels, with an estimated 570 million cases projected by 2025 (1). Prediabetes, the precursor stage of diabetes, is primarily characterized by impaired fasting glucose (IFG) and impaired glucose tolerance (IGT). The population affected by prediabetes continues to grow (2). By 2030, the number of individuals with prediabetes is expected to exceed 470 million (3) Notably, the annual conversion rate from prediabetes to diabetes ranges from approximately 5% to 10% (4). Moreover, diabetes significantly shortens life expectancy. The World Health Organization (WHO) predicts that by 2030, diabetes will become the seventh leading cause of death globally (5). Compared to individuals with normal glucose metabolism, patients with diabetes or prediabetes (hereinafter referred to as diabetes/prediabetes) face significantly increased risks for macrovascular (6–8) and microvascular complications (9). Consequently, cardiovascular disease (CVD) a leading cause of mortality and disability among diabetes patients (10). Effectively managing diabetes or prediabetes to reduce mortality risk presents a formidable challenge.

Among the numerous factors influencing blood glucose levels, obesity is undoubtedly one of the most significant. The prevalence of diabetes/prediabetes in the United States is rising alongside obesity. Most patients with diabetes/prediabetes exhibit excessive adipose tissue, which stimulates inflammatory responses and immune dysfunction, serving as key contributors to insulin resistance (11). Although Body Mass Index (BMI) is a widely accepted standard for assessing obesity, it is inadequate for evaluating visceral fat, dyslipidemia, and insulin resistance. Consequently, several new anthropometric tools have been developed to better reflect these characteristics. For example, the abdominal obesity index (ABSI) (12), lipid accumulation product (LAP), and visceral adiposity index (VAI) (13) are considered effective new indicators for predicting diabetes risk compared to BMI (14). Additionally, the triglyceride-glucose index (TyG) (15) has advantages, such as not requiring highly precise insulin levels and overcoming poor measurement reproducibility. It is regarded as an effective alternative to traditional insulin resistance indicators, such as HOMA-IR and QUICKI (16). This advancement overcomes the limitations of traditional indicators in clinical practice (17). Furthermore, several studies have developed novel indices based on TyG by incorporating various anthropometric measurements. Examples include the triglyceride glucose-body mass index (TyG-BMI), triglyceride glucose-waist-to-height ratio (TyG-WHtR), triglyceride glucose-weight-adjusted waist circumference(TyG-WWI) and triglyceride glucose-waist circumference (TyG-WC) (18). These indices are also considered effective tools for predicting diabetes risk. Despite the varying degrees of potential these indicators have shown in predicting diabetes, there is currently no consensus on their effectiveness in predicting mortality risk among patients with diabetes/prediabetes.

This study aims to explore the predictive capabilities of obesity and lipid-related indices (TyG, TyG-BMI, TyG-WHtR, TyG-WWI, TyG-WC, LAP, VAI, and ABSI) for all-cause and cardiovascular mortality among patients with diabetes/prediabetes, utilizing the National Health and Nutrition Examination Survey (NHANES) database. Additionally, we will compare the predictive abilities of these indices using machine learning models to identify the most accurate predictive factors.

## Materials and methods

### Study population in NHANES

**Figure 1:**
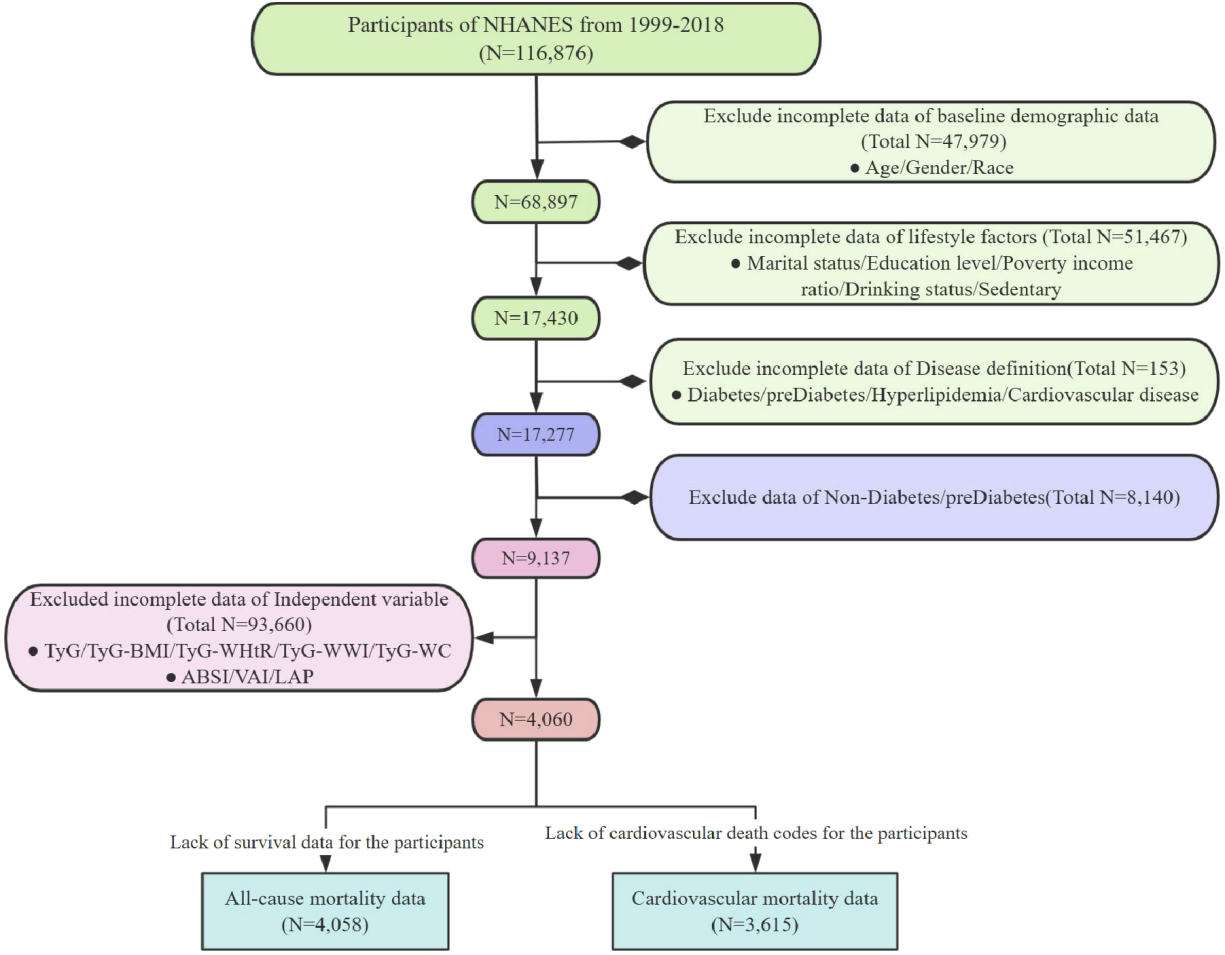
Flowchart of inclusion and exclusion criteria for the study.

In this study, we analyzed data collected from 1999 to 2018. The criteria for excluding samples included the following (1): lack of necessary parameters for assessing obesity and lipid-related indices; (2) absence of definitional information for diabetes and prediabetes; (3) missing covariate data; (4) absence of survival data (**Figure 1**). The NHANES study protocol was approved by the Institutional Review Board of the National Center for Health Statistics (NCHS), and all participants provided written informed consent. For more detailed information about this study, please visit: www.cdc.gov/nchs/nhanes/irba98.htm.

### Assessment of the diagnosis of prediabetes and diabetes

The diagnosis of diabetes was based on one or more of the following criteria: (1) a medical diagnosis confirmed by the patient’s healthcare provider with self-reporting; (2) glycated hemoglobin (HbA1c) level ≥ 6.5%; (3) FPG level ≥ 7.0 mmol/L; (4) questionnaire results indicating that the patient is using diabetes medications. Prediabetes was defined by the following criteria: (1) a diagnosis confirmed by a healthcare professional through self-reporting; (2) HbA1c levels between 5.7% and less than 6.5%; (3) FPG levels between 100 mg/dL and 125 mg/dL (19).

### Definitions of obesity and lipid-related indices

In this study, the obesity and lipid-related indices included TyG, TyG-BMI, TyG-WWI, TyG-WHtR, TyG-WC, ABSI, LAP, and VAI. The calculation methods for TyG and its related obesity indicators are as follows. Since ABSI values are typically low, the values presented in this study are shown as 10-fold multiples (18):


$$\text{TyG}=1\text{n}\left(\frac{\text{Triglyceride}(\text{mg}/\text{dL})\times \text{FPG}(\text{mg}/\text{dL})}{2}\right)$$

$$\text{TyG}-\text{WHtR}=\text{TyG}\times \frac{\text{WC}(\text{cm})}{\text{Height}(\text{m})}$$

$$\text{TyG}-\text{BMI}=\text{TyG}\times \frac{\text{Weight}(\text{kg})}{\text{Heigh}{\text{t}}^{2}({\text{m}}^{2})}$$

$$\text{TyG}-\text{WWI}=\text{TyG}\times \frac{\text{WC}(\text{cm})}{\sqrt{\text{Weight}(\text{kg})}}$$

TyG−WC=TyG×WC

VAI (13) is calculated as follows:


$$\begin{array}{c} \text{Males}:\text{VAI}=\frac{\text{WC}(\text{cm})}{39.68+(1.88\times \text{BMI}(\text{kg}/{\text{m}}^{2}))}\times \left(\frac{\text{Triglyceride}(\text{mmol}/\text{L})}{1.03}\right)\times \left(\frac{1.31}{\text{HDL}-\text{C}(\text{mmol}/\text{L})}\right)\\ \text{Females}:\text{VAI}=\frac{\text{WC}(\text{cm})}{36.58+(1.89\times \text{BMI}(\text{kg}/{\text{m}}^{2}))}\times \left(\frac{\text{Triglyceride}(\text{mmol}/\text{L})}{0.81}\right)\times \left(\frac{1.52}{\text{HDL}-\text{C}(\text{mmol}/\text{L})}\right) \end{array}$$

LAP (13) is calculated as follows:


$$\begin{array}{c} \text{Males}:\text{LAP}=(\text{WC}(\text{cm})-65)\times \text{Triglyceride}(\text{mmol}/\text{L})\\ \text{Female}:\text{LAP}=(\text{WC}(\text{cm})-58)\times \text{Triglyceride}(\text{mmol}/\text{L}) \end{array}$$

The ABSI (12) is calculated as follows:


$$\text{ABSI}=\frac{\text{WC}(\text{cm})}{\text{BMI}{\left(\text{kg}/{\text{m}}^{2}\right)}^{\frac{2}{3}}\times \text{Height}{\left(\text{m}\right)}^{\frac{1}{2}}}$$

FPG: Fasting blood glucose; HDL-C: High density lipoprotein cholesterol; WWI: Weight-adjusted waist circumference index; WC: Waist circumference.

### Mortality

In this study, mortality data for all NHANES participants were matched with the National Death Index (NDI) using probabilistic matching methods, with a cutoff date of December 31, 2019. This process was used to calculate all-cause mortality rates. Additionally, considering the close relationship between obesity, lipid-related indices, and diabetes in relation to cardiovascular mortality risk, we used the death codes provided by the NDI to ascertain the cause of death. Causes of death were classified as cardiovascular-related according to the International Classification of Diseases, Tenth Revision (ICD-10), using relevant codes I00-I09, I11, I13, and I20-I51.

### Covariates

Covariate information for this study was collected from NHANES demographic data, questionnaires, and laboratory tests. We categorized the covariates into three main groups: baseline demographic data (age, gender, race), lifestyle factors (marital status, education level, BMI, alcohol consumption, and sedentary behavior), and comorbidities (hyperlipidemia, and CVD). The specific definitions are as follows:

The original five racial classifications from NHANES were condensed into three categories: Hispanic, Non-Hispanic Black, and Non-Hispanic White and Other; Marital status was categorized as divorced, married, or unmarried; Education level was classified as Below high school (Less Than 9th Grade), High school graduate or GED (9-11th Grade, including 12th grade with no diploma), and Some college or above (Some College or AA degree/College Graduate or above); BMI classifications were defined as normal (<25 kg/m²), obese (≥30 kg/m²), and overweight (≥25 kg/m² but <30 kg/m²); Sedentary behavior was defined as sitting or reclining for more than 480 minutes per day, or responding the questionnaire with an emphasis on a sedentary typical day; Alcohol consumption status was classified into three categories: current drinker (defined as having consumed more than 12 types of alcoholic beverages in their lifetime and currently consuming), former drinker (defined as having consumed more than 12 types of alcoholic beverages at any time during their lifetime but not in the past year), and never drinker (defined as having consumed no more than 12 types of alcoholic beverages in their lifetime); Hyperlipidemia was defined by any of the following criteria: total cholesterol levels equal to or exceeding 200 mg/dL, triglyceride levels equal to or exceeding 150 mg/dL, male HDL-C levels below 40 mg/dL, female HDL-C levels below 50 mg/dL, or low-density lipoprotein cholesterol (LDL-C) levels equal to or exceeding 130 mg/dL; CVD was defined as a positive response to any of the following questions: “Has a doctor or other health professional ever told you that you have congestive heart failure (CHF), coronary heart disease (CHD), angina, a heart attack, or a stroke?”

### Statistical analysis

We employed a complex sampling design to ensure nationally representative estimates, and all analyses were adjusted for survey design and weighting variables. The new sample weights were calculated by dividing the original two-year sample weights by 20. Continuous variables are presented as means ± standard deviation (SD), while categorical variables are expressed as counts (N) and percentages (%). The obesity and lipid-related indices were categorized into four groups using quartiles. We used weighted t-tests (for continuous variables) or weighted chi-square tests (for categorical variables) to assess differences between survival and mortality group. The survival probabilities of diabetes/prediabetes patients under different obesity and lipid-related indices were compared using Kaplan-Meier (KM) curves and log-rank tests. The Cox regression model was used to analyze the mortality risk in diabetes/prediabetes patients, with model construction undergoing multiple adjustments: Model 1 adjusted for baseline demographic data; Model 2 further adjusted for lifestyle factors; and Model 3 adjusted for comorbidities on top of Model 2. A p-value of less than 0.05 was considered statistically significant for all two-sided tests. Furthermore, we employed a restricted cubic spline (RCS) model to treat obesity and lipid-related indices as continuous variables, investigating the linear and non-linear associations between these indices and mortality risk in diabetes/prediabetes patients by setting the 10th, 50th, and 90th percentiles as nodes of the RCS (20).

### Machine learning

This study also employed machine learning (ML) modeling strategies to compare predictive abilities of obesity and lipid-related indices for mortality risk based on the Cox regression model. We used supervised MLs, integrating various obesity and lipid-related indices, components of each index, all covariates, and survival data into the machine learning dataset (21): extreme gradient boosting (XGBoost), decision tree (DT), robust support vector machine (RSVM), elastic net regression (Enet), multi-layer perceptron (MLP), logistic regression, random forest (RF), and k-nearest neighbors (KNN). The dataset was divided into two non-overlapping parts: a training set (60%) and a testing set (40%). In the training dataset, each model underwent automatic hyperparameter tuning using Bayesian optimization and five-fold cross-validation. When comparing the eight machine learning algorithms, we synthesized the assessment of the best algorithm using the receiver operating characteristic - area under the curve (ROC-AUC), accuracy, precision, recall, and calibration curves. We subsequently applied SHapley Additive Explanations (SHAP) to interpret the machine learning models, aiming to address the black box issue associated with these models. The Shapley value, derived from cooperative game theory, quantifies the importance of each feature in the model by calculating marginal contributions (22). We used the “fastshap” package to generate SHAP beeswarm plots to visualize each variable’s contribution to individual predictions. This clearly illustrates the significance of obesity and lipid-related indices and analyzes how the components of different indices contribute to and influence mortality risk.

## Results

### Baseline characteristics of study participants

This study included 4,058 participants with diabetes/prediabetes, of whom 640 (12%) died before December 31, 2019. Significant differences were observed between the mortality group and the survival group across multiple variables. First, the mortality group had a higher average age of 70.5 years and relatively fewer male survivors. Additionally, the mortality group had a higher proportion of Non-Hispanic White and other racial groups, as well as a greater proportion of married individuals. Notably, the mortality group exhibited higher rates of sedentary behavior, poverty, and low educational attainment, along with higher proportions of non-drinkers, individuals with hyperlipidemia, and CVD. Furthermore, some anthropometric measures, such as height, weight, and waist circumference, were slightly lower in the mortality group compared to the survival group. However, new anthropometric measurements derived from these indicators, such as the WWI and ABSI, were higher in the mortality group. Blood lipid and glucose levels were also significantly elevated in the mortality group. Additionally, the TyG was higher in the mortality group. Other derived anthropometric indices, such as TyG-WHtR and TyG-WWI, were also elevated compared to the survival group, with the VAI significantly higher as well. However, no significant difference was found in the LAP between the two groups (**Table 1**).

**Table 1:** Characteristics of participants according to All-cause mortality. (NHANES 1999-2018, N = 4,058).

Characteristic	Overall, N = 4058 (100%)^1,2^	Survival Group, N = 3418 (88%)^1,2^	Mortality Group, N = 640 (12%)^1,2^	P Value
Age (years)	54.6 (16.5)	52.4 (15.8)	70.5 (11.9)	<0.001
Sex				0.036
*Female*	2,470 (58%)	2,078 (58%)	392 (63%)	
*Male*	1,588 (42%)	1,340 (42%)	248 (37%)	
Race				<0.001
*Non-Hispanic White and Other*	2,033 (72%)	1,644 (71%)	389 (80%)	
*Hispanic*	1,085 (15%)	967 (16%)	118 (7.3%)	
*Non-Hispanic Black*	940 (13%)	807 (13%)	133 (13%)	
Marital				0.014
*Divorced*	2,232 (59%)	1,923 (60%)	309 (53%)	
*Married*	1,677 (39%)	1,365 (38%)	312 (45%)	
*Never married*	149 (2.6%)	130 (2.6%)	19 (2.3%)	
PIR				<0.001
*High(>3.49)*	1,040 (36%)	932 (38%)	108 (22%)	
*Medium(>1.39,<=3.49)*	1,573 (38%)	1,302 (37%)	271 (45%)	
*Low(≤1.39)*	1,445 (26%)	1,184 (25%)	261 (33%)	
Sedentary				0.001
*Non Sedentary*	2,895 (69%)	2,499 (73%)	396 (62%)	
*Sedentary*	1,163 (31%)	919 (27%)	244 (38%)	
Education				<0.001
*Below high school*	599 (8.4%)	456 (7.5%)	143 (15%)	
*High school graduate or GED*	1,611 (40%)	1,319 (39%)	292 (50%)	
*Some college or above*	1,848 (51%)	1,643 (54%)	205 (35%)	
Weight(Kg)	86 (23)	87 (23)	79 (21)	<0.001
Height(Cm)	166 (10)	167 (10)	164 (10)	<0.001
Waist circumference	104 (17)	104 (17)	102 (16)	0.029
BMI	31 (7)	31 (7)	29 (7)	<0.001
*Normal(≥18.5,<25)*	799 (19%)	633 (18%)	166 (27%)	
*Obese(≥30)*	1,935 (49%)	1,678 (50%)	257 (40%)	
*Overweight(≥25,<30)*	1,292 (31%)	1,084 (31%)	208 (32%)	
Drinking status				<0.001
*Current drinker*	1,538 (45%)	1,411 (48%)	127 (22%)	
*Former drinker*	1,095 (25%)	867 (23%)	228 (35%)	
*Never drinker*	1,425 (30%)	1,140 (28%)	285 (42%)	
Hyperlipidemia				0.027
*Hyperlipidemia*	3,266 (80%)	2,729 (80%)	537 (84%)	
*Non-Hyperlipidemia*	792 (20%)	689 (20%)	103 (16%)	
CVD				<0.001
*CVD*	625 (13%)	421 (12%)	204 (32%)	
*Non-CVD*	3,433 (87%)	2,997 (88%)	436 (68%)	
Insulin	16 (18)	16 (18)	15 (18)	0.4
Triglyceride	128 (67)	125 (66)	150 (70)	<0.001
Blood glucose	118 (38)	117 (35)	128 (52)	<0.001
WHtR	0.63 (0.10)	0.63 (0.10)	0.63 (0.09)	0.8
WWI	11.30 (0.80)	11.26 (0.80)	11.61 (0.78)	<0.001
TyG-BMI	273 (71)	274 (72)	264 (67)	0.019
TyG-WHtR	5.51 (1.05)	5.50 (1.05)	5.65 (0.99)	0.008
TyG-WWI	99 (11)	98 (11)	105 (11)	<0.001
TyG-WC	916 (174)	915 (175)	925 (167)	0.3
TyG	8.76 (0.62)	8.73 (0.62)	9.01 (0.59)	<0.001
LAP	214 (92)	215 (92)	209 (87)	0.2
ABSI	0.82 (0.05)	0.82 (0.05)	0.85 (0.05)	<0.001
VAI	2.14 (1.53)	2.08 (1.49)	2.59 (1.78)	<0.001

^1^Mean ± SD for continuous; n (%) for categorical.

^2^t-test adapted to complex survey samples; chi-squared test with Rao & Scott’s second-order correction.

triglyceride glucose-waist circumference.

TyG, Triglyceride Glucose; TyG-BMI, Triglyceride Glucose - Body Mass Index; TyG-WHtR, Triglyceride Glucose - Waist to Height Ratio; TyG-WWI, Triglyceride Glucose - Weight Adjusted Waist Index; TyG-WC, Triglyceride Glucose - Waist Circumference; ABSI, A Body Shape Index; LAP, Lipid Accumulation Product; VAI, Visceral Adiposity Index.

### Survival patterns of diabetes/prediabetes patients by quartile levels of obesity and lipid-related indices

**Figure 2:**
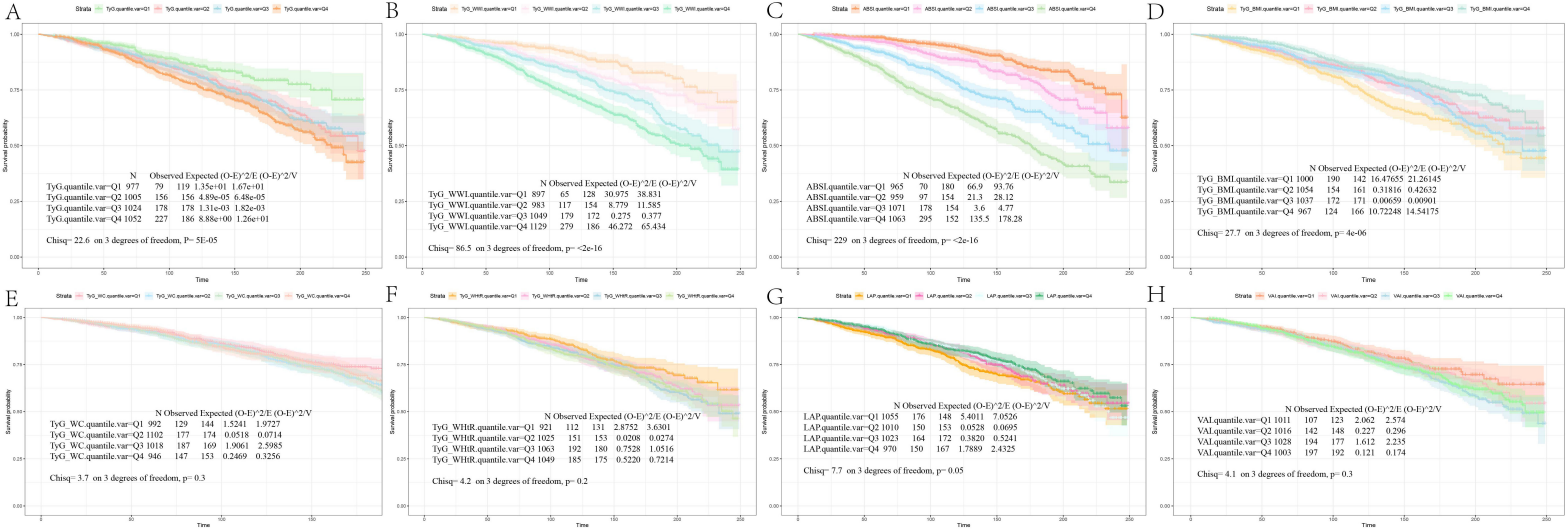
Kaplan-Meier survival analysis curves for all-Cause mortality. The Kaplan-Meier curves show the cumulative probabilities of all-cause mortality at 250 days for each group: **(A)** TyG: Q1 (6.56, 8.33), Q2 (8.34, 8.74), Q3 (8.75, 9.17), Q4 (9.18, 11.03); **(B)** TyG-WWI: Q1 (60.85, 90.83), Q2 (90.84, 98.38), Q3 (98.39, 106.25), Q4 (106.26, 144.15); **(C)** ABSI: Q1 (0.60, 0.78), Q2 (0.79, 0.81), Q3 (0.82, 0.85), Q4 (0.86, 0.99); **(D)** TyG-BMI: Q1 (115.40, 223.37), Q2 (223.38, 262.31), Q3 (262.32, 313.23), Q4 (313.24, 620.83); **(E)** TyG-WC: Q1 (453.12, 792.26), Q2 (792.27, 902.24), Q3 (902.25, 1025.15), Q4 (1025.16, 1648.81); **(F)** TyG-WHtR: Q1 (2.63, 4.73), Q2 (4.74, 5.40), Q3 (5.41, 6.14), Q4 (6.15, 10.23); **(G)** LAP: Q1 (3.07, 149.93), Q2 (149.94, 202.77), Q3 (202.78, 268.45), Q4 (268.46, 604.96); **(H)** VAI: Q1 (0.15, 1.01), Q2 (1.02, 1.68), Q3 (1.69, 2.75), Q4 (2.76, 11.39). TyG, Triglyceride Glucose; TyG-BMI, Triglyceride Glucose - Body Mass Index; TyG-WHtR, Triglyceride Glucose - Waist to Height Ratio; TyG-WWI, Triglyceride Glucose - Weight Adjusted Waist Index; TyG-WC, Triglyceride Glucose - Waist Circumference; ABSI, A Body Shape Index; LAP, Lipid Accumulation Product; VAI, Visceral Adiposity Index.

We conducted a survival analysis on the indices that demonstrated statistical differences between the survival and mortality groups in **Table 1**. The KM curves revealed that diabetes/prediabetes patients in the lowest quartile of TyG, TyG-WWI, and ABSI had significantly higher overall survival probabilities compared to those in the highest quartile (P = 5e-05, P < 2e-16, and P < 2e-16, respectively) (**Figures 2A–C**). Additionally, the TyG-BMI in the Q2 group demonstrated the highest survival probability (P = 4e-06) (**Figure 2D**), while TyG-WC, TyG-WHtR, LAP, and VAI did not show significant differences (P = 0.3, P = 0.2, P = 0.05, and P = 0.3, respectively) (**Figures 2E–H**).

### Associations between obesity and lipid-related indices and mortality

**Figure 3:**
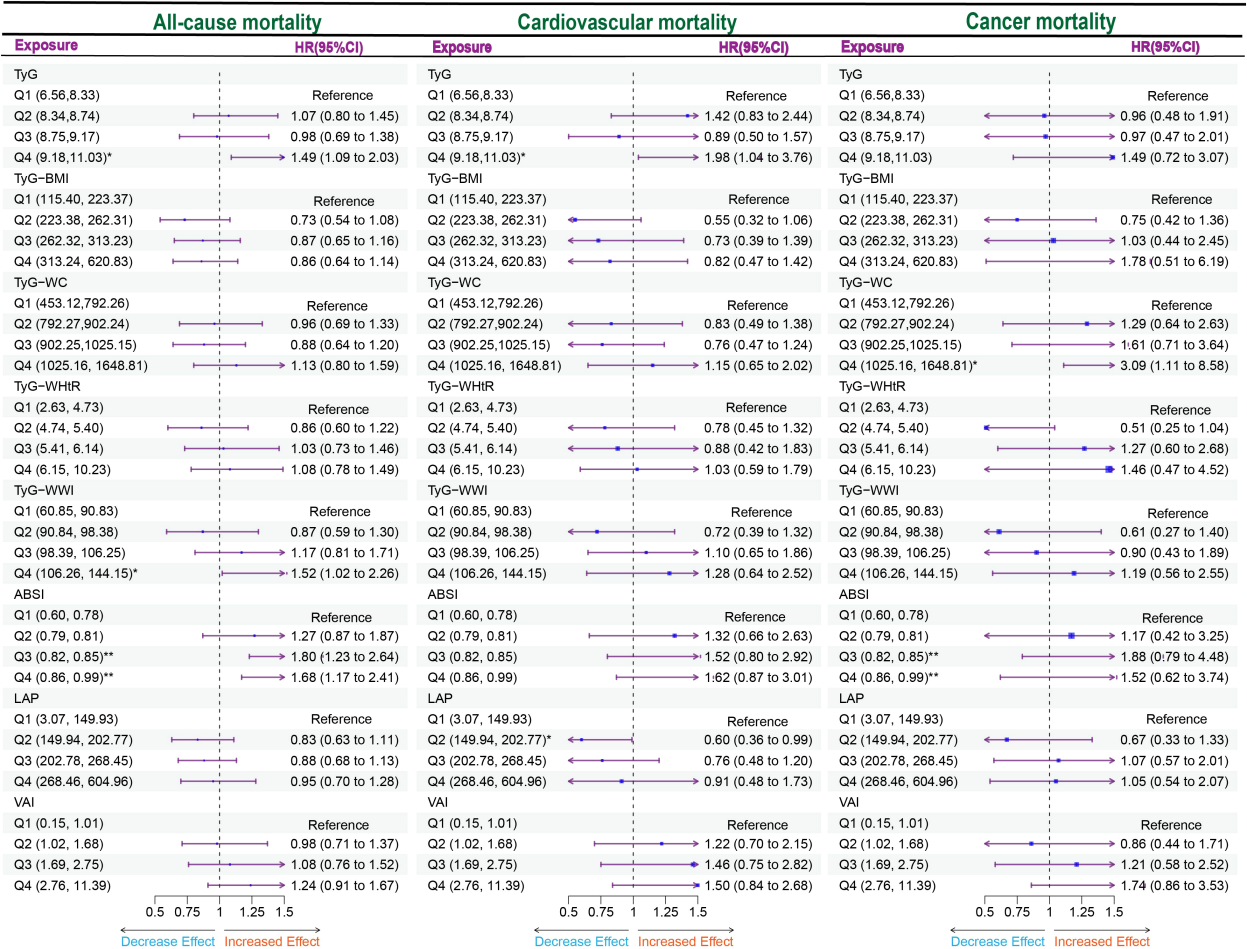
Forest plot of obesity and lipid-related indicators with mortality. Multiple Cox regression model: Model 1: Adjusted for Age, Gender, Race; Model 2: Adjusted for Age, Gender, Race, Education, Marital, PIR, Sedentary, Drinking status; Model 3: Adjusted for Age, Gender, Race, Education, Marital, PIR, Sedentary, Drinking status, Hyperlipidemia, CVD. TyG, Triglyceride Glucose; TyG-BMI, Triglyceride Glucose - Body Mass Index; TyG-WHtR, Triglyceride Glucose - Waist to Height Ratio; TyG-WWI, Triglyceride Glucose - Weight Adjusted Waist Index; TyG-WC, Triglyceride Glucose - Waist Circumference; ABSI, A Body Shape Index; LAP, Lipid Accumulation Product; VAI, Visceral Adiposity Index. *P < 0.05; **P < 0.01.

We performed a quartile-based analysis of obesity and various lipid-related indices, including TyG, TyG-BMI, TyG-WHtR, TyG-WWI, TyG-WC, LAP, VAI, and ABSI. The results from the Cox regression analysis indicated significant associations between TyG, TyG-WWI, and ABSI and all-cause mortality in diabetes/prediabetes patients. After adjustment in Model 3, compared to baseline levels (Q1), the highest quartile of TyG (Q4: 9.18, 11.03) and TyG-WWI (Q4: 106.26, 144.15) respectively increased the risk of all-cause mortality, with a HR of 1.49 (95% CI: 1.09-2.03) and 1.52 (95% CI: 1.02-2.26). Furthermore, ABSI in Q3 (0.82, 0.85) and Q4 (0.86, 0.99) also indicated increased all-cause mortality risk, with HRs of 1.80 (95% CI: 1.23-2.64) and 1.68 (95% CI: 1.17-2.41), respectively. Additionally, the analysis of ungrouped continuous variables revealed that for each one-unit increase in TyG, TyG-WWI, and ABSI, the all-cause mortality risk increased by 1.4 times, 1.02 times, and 48.6 times, respectively (**Figure 3**, **Supplementary Table S2**).

Moreover, focusing solely on patients who died from cardiovascular causes (**Supplementary Table S1**), the Cox regression analysis indicated a significant association between TyG and cardiovascular mortality in diabetes/prediabetes patients. After adjustment in Model 3, the highest quartile of TyG (Q4: 8.75, 11.03) was associated with an increased risk of cardiovascular mortality, with a HR of 1.98 (95% CI: 1.04-2.35). Additionally, the analysis of ungrouped continuous variables revealed that for each one-unit increase in TyG, the risk of cardiovascular mortality increased by 1.57 times (**Figure 3**, **Supplementary Table S3**). Additionally, TyG-WC in the Q4 was associated with elevated cancer-related mortality, with a HR of 3.09 (95% CI: 1.11–8.58) (**Figure 3**, **Supplementary Table S4**).

### Race differences in analysis

Cox regression analysis of race revealed that race-specific associations between obesity/lipid-related indicators and mortality risks. For Hispanic populations, elevated TyG quartiles (Q4) significantly increased all-cause mortality (HR = 2.35, 95% CI: 1.18–4.68) and cardiovascular mortality (HR = 2.42, 95% CI: 1.12–5.24), with per-unit TyG increases further amplifying risks (all-cause: HR = 1.94; Cardiovascular mortality: HR = 1.69). Non-Hispanic Black groups exhibited extreme obesity-driven risks, particularly with higher ABSI quartiles (all-cause mortality Q2–Q4 HRs = 3.79–3.00, all *P < 0.05; cardiovascular mortality per-unit HR = 49.3, 95% CI: 6.19–392, ***P < 0.001). Additionally, ABSI at the Q4 level demonstrated an elevated risk of all-cause mortality in both the Hispanic and Non-Hispanic White and Other groups. Non-Hispanic White/Other populations only showed significant cancer mortality risks with TyG-WC Q4 (HR = 3.26, 95% CI: 1.03–10.34). LAP/VAI showed no significant associations across races (**Tables 2**–**4**).

**Table 2:** Subgroup analysis of all-cause mortality risk based on race.

All-cause mortality	Hispanic	Non-Hispanic Black	Non-Hispanic White and Other
TyG			
Q1 (6.56,8.33)	Reference	Reference	Reference
Q2 (8.34,8.74)	1.66 (0.94,2.93)	0.64 (0.25,1.62)	0.95 (0.65,1.40)
Q3 (8.75,9.17)	1.73 (0.98,3.03)	0.56 (0.22,1.43)	0.88 (0.57,1.37)
Q4 (9.18,11.03)	2.35 (1.18,4.68)*	0.70 (0.29,1.68)	1.39 (0.94,2.03)
TyG(Per 1 unit increase)	1.94 (1.34,2.79)***	1.20 (0.81,1.76)	1.36 (1.09,1.70)**
TyG-BMI			
Q1 (115.40, 223.37)	Reference	Reference	Reference
Q2 (223.38, 262.31)	0.75 (0.38,1.48)	0.31 (0.13,0.77)*	0.87 (0.58,1.29)
Q3 (262.32, 313.23)	1.19 (0.60,2.35)	0.55 (0.23,1.32)	1.04 (0.61,1.78)
Q4 (313.24, 620.83)	2.22 (0.85,5.78)	0.73 (0.19,2.76)	1.01 (0.45,2.28)
TyG-BMI(Per 1 unit increase)	1.02 (1.01,1.03)***	1.01 (0.99,1.02)	1.01 (1.00,1.02)*
TyG-WC			
Q1 (453.12, 792.26)	Reference	Reference	Reference
Q2 (792.27, 902.24)	0.95 (0.50,1.78)	0.77 (0.28,2.06)	1.18 (0.77,1.80)
Q3 (902.25, 1025.15)	1.48 (0.73,2.97)	0.88 (0.34,2.31)	1.12 (0.69,1.82)
Q4 (1025.16, 1648.81)	2.24 (1.03,4.89)*	2.25 (0.59,8.55)	1.68 (0.83,3.39)
TyG-WC(Per 1 unit increase)	1.00 (1.00, 1.01)***	1.00 (1.00,1.01)	1.00 (1.00, 1.00)
TyG-WHtR			
Q1 (2.63, 4.73)	Reference	Reference	Reference
Q2 (4.74, 5.40)	1.05 (0.52,2.11)	1.01 (0.32,3.20)	1.01 (0.66,1.56)
Q3 (5.41, 6.14)	1.53 (0.76,3.07)	0.71 (0.26,1.93)	1.47 (0.88,2.45)
Q4 (6.15, 10.23)	2.79 (1.24,6.24)*	1.76 (0.53,5.81)	1.66 (0.92,2.99)
TyG-WHtR(Per 1 unit increase)	2.24 (1.59,3.17)***	1.42 (0.89,2.26)	1.45 (1.13,1.86)**
TyG-WWI			
Q1 (60.85, 90.83)	Reference	Reference	Reference
Q2 (90.84, 98.38)	1.29 (0.69,2.40)	0.87 (0.20,3.69)	0.84 (0.50,1.42)
Q3 (98.39, 106.25)	2.07 (1.08,3.94)*	1.15 (0.24,5.58)	1.21 (0.72,2.01)
Q4 (106.26, 144.15)	2.22 (1.08,4.56)*	1.43 (0.29,7.01)	1.68 (0.99,2.86)
TyG-WWI(Per 1 unit increase)	1.05 (1.03,1.06)***	1.02 (0.99,1.05)	1.02 (1.01,1.04)**
ABSI			
Q1 (0.60, 0.78)	Reference	Reference	Reference
Q2 (0.79, 0.81)	1.60 (0.91,2.80)	3.79 (1.71,8.42)**	1.13 (0.65,1.95)
Q3 (0.82, 0.85)	1.89 (0.99,3.61)	2.11 (1.00,4.43)*	1.78 (1.09,2.91)*
Q4 (0.86, 0.99)	2.80 (1.56,5.03)***	3.00 (1.20,7.48)*	1.51 (1.05,1.97)*
ABSI(Per 1 unit increase)	56.1 (6.61, 476)***	49.3 (6.19, 392)***	27.83 (11.89,43.77)*
LAP			
Q1 (3.07, 149.93)	Reference	Reference	Reference
Q2 (149.94, 202.77)	0.66 (0.33,1.32)	0.54 (0.22,1.28)	0.98 (0.67,1.42)
Q3 (202.78, 268.45)	0.83 (0.43,1.62)	0.48 (0.22,1.06)	1.10 (0.77,1.59)
Q4 (268.46, 604.96)	1.16 (0.54,2.49)	0.60 (0.20,1.86)	1.21 (0.70,2.10)
LAP(Per 1 unit increase)	1.00 (1.00,1.01)	1.00 (0.99,1.00)	1.00 (1.00, 1.00)
VAI			
Q1 (0.15, 1.01)	Reference	Reference	Reference
Q2 (1.02, 1.68)	1.17 (0.69,1.98)	0.76 (0.26,2.21)	0.96 (0.65,1.43)
Q3 (1.69, 2.75)	1.60 (0.91,2.84)	1.35 (0.43,4.25)	0.99 (0.65,1.50)
Q4 (2.76, 11.39)	1.54 (0.88,2.72)	1.25 (0.41,3.82)	1.18 (0.82,1.68)
VAI(Per 1 unit increase)	1.24 (1.09,1.40)***	1.08 (0.94,1.24)	1.06 (0.99,1.13)

*P < 0.05; **P < 0.01.

Multiple Cox regression model 3: Adjusted for Age, Sex, Education, Marital, PIR, Sedentary, Drinking status, Hyperlipidemia, CVD.

**Table 3:** Subgroup analysis of cardiovascular risk based on race.

Cardiovascular mortality	Hispanic	Non-Hispanic Black	Non-Hispanic White and Other
TyG			
Q1 (6.56,8.33)	Reference	Reference	Reference
Q2 (8.34,8.74)	1.49 (0.70,3.14)	0.58 (0.08,3.98)	1.49 (0.70,3.14)
Q3 (8.75,9.17)	0.89 (0.42,1.87)	0.41 (0.07,2.55)	0.89 (0.42,1.87)
Q4 (9.18,11.03)	2.42 (1.12,5.24)*	0.47 (0.09,2.41)	2.42 (1.12,5.24)*
TyG(Per 1 unit increase)	1.69 (1.05,2.72)*	0.98 (0.41,2.38)	1.69 (1.05,2.72)*
TyG-BMI			
Q1 (115.40, 223.37)	Reference	Reference	Reference
Q2 (223.38, 262.31)	0.62 (0.29,1.31)	0.07 (0.02,0.23)***	0.62 (0.29,1.31)
Q3 (262.32, 313.23)	0.60 (0.16,2.27)	0.25 (0.07,0.93)*	0.60 (0.16,2.27)
Q4 (313.24, 620.83)	0.64 (0.10,4.12)	0.27 (0.03,2.71)	0.64 (0.10,4.12)
TyG-BMI(Per 1 unit increase)	1.02 (1.00,1.03)*	1.00 (0.97,1.03)	1.02 (1.00,1.03)*
TyG-WC			
Q1 (453.12, 792.26)	Reference	Reference	Reference
Q2 (792.27, 902.24)	0.94 (0.48,1.86)	0.67 (0.15,2.98)	0.94 (0.48,1.86)
Q3 (902.25, 1025.15)	0.82 (0.34,1.94)	0.81 (0.24,2.73)	0.82 (0.34,1.94)
Q4 (1025.16, 1648.81)	1.40 (0.36,5.47)	3.78 (0.76,18.72)	1.40 (0.36,5.47)
TyG-WC(Per 1 unit increase)	1.00 (1.00,1.00)	1.00 (1.00,1.01)	1.00(1.00, 1.00)
TyG-WHtR			
Q1 (2.63, 4.73)	Reference	Reference	Reference
Q2 (4.74, 5.40)	0.71 (0.35,1.45)	2.88 (0.64,12.93)	0.71 (0.35,1.45)
Q3 (5.41, 6.14)	1.01 (0.34,3.02)	0.35 (0.06,1.94)	1.01 (0.34,3.02)
Q4 (6.15, 10.23)	1.15 (0.37,3.63)	4.52 (0.67,30.32)	1.15 (0.37,3.63)
TyG-WHtR(Per 1 unit increase)	1.36 (0.85,2.17)	1.50 (0.65,3.48)	1.36 (0.85,2.17)
TyG-WWI			
Q1 (60.85, 90.83)	Reference	Reference	Reference
Q2 (90.84, 98.38)	0.58 (0.28,1.19)	1.41 (0.18,10.99)	0.58 (0.28,1.19)
Q3 (98.39, 106.25)	0.98 (0.49,1.93)	1.04 (0.10,10.56)	0.98 (0.49,1.93)
Q4 (106.26, 144.15)	1.26 (0.54,2.98)	2.01 (0.27,14.72)	1.26 (0.54,2.98)
TyG-WWI(Per 1 unit increase)	1.02 (0.99,1.05)	1.02 (0.98,1.07)	1.02 (0.99,1.05)
ABSI			
Q1 (0.60, 0.78)	Reference	Reference	Reference
Q2 (0.79, 0.81)	1.20 (0.50,2.88)	2.72 (0.33,22.09)	1.20 (0.50,2.88)
Q3 (0.82, 0.85)	1.21 (0.57,2.57)	2.96 (0.31,28.48)	1.21 (0.57,2.57)
Q4 (0.86, 0.99)	1.16 (0.56,2.40)	4.84 (0.56,42.09)	1.16 (0.56,2.40)
ABSI(Per 1 unit increase)	1.75 (0.04,77.20)	49.3(6.19, 392)***	1.75 (0.04,77.20)
LAP			
Q1 (3.07, 149.93)	Reference	Reference	Reference
Q2 (149.94, 202.77)	0.69 (0.36,1.35)	0.26 (0.06,1.07)	0.69 (0.36,1.35)
Q3 (202.78, 268.45)	0.87 (0.42,1.78)	0.31 (0.10,0.98)*	0.87 (0.42,1.78)
Q4 (268.46, 604.96)	1.00 (0.27,3.64)	0.74 (0.13,4.22)	1.00 (0.27,3.64)
LAP(Per 1 unit increase)	1.00 (1.00,1.01)	0.99 (0.98,1.00)	1.00(1.00, 1.01)
VAI			
Q1 (0.15, 1.01)	Reference	Reference	Reference
Q2 (1.02, 1.68)	1.36 (0.68,2.72)	0.77 (0.13,4.53)	1.36 (0.68,2.72)
Q3 (1.69, 2.75)	1.58 (0.71,3.49)	1.41 (0.29,6.79)	1.58 (0.71,3.49)
Q4 (2.76, 11.39)	1.62 (0.84,3.12)	0.72 (0.14,3.63)	1.62 (0.84,3.12)
VAI(Per 1 unit increase)	1.06 (0.94,1.20)	0.98 (0.72,1.33)	1.06 (0.94,1.20)

*P < 0.05; **P < 0.01.

Multiple Cox regression model 3: Adjusted for Age, Sex, Education, Marital, PIR, Sedentary, Drinking status, Hyperlipidemia, CVD.

**Table 4:** Subgroup analysis of cancer mortality risk based on race.

Cancer mortality	Hispanic	Non-Hispanic Black	Non-Hispanic White and Other
TyG			
Q1 (6.56,8.33)	Reference	Reference	Reference
Q2 (8.34,8.74)	0.54 (0.23,1.28)	2.93 (0.23,36.88)	4.42 (0.90,21.67)
Q3 (8.75,9.17)	0.65 (0.28,1.51)	2.96 (0.25,35.12)	3.67 (0.72,18.81)
Q4 (9.18,11.03)	1.17 (0.52,2.65)	1.83 (0.13,25.85)	3.86 (0.56,26.56)
TyG(Per 1 unit increase)	1.33 (0.76,2.32)	1.08 (0.50,2.34)	1.62 (0.76,3.47)
TyG-BMI			
Q1 (115.40, 223.37)	Reference	Reference	Reference
Q2 (223.38, 262.31)	0.89 (0.42,1.90)	1.44 (0.37,5.59)	0.46 (0.10,2.17)
Q3 (262.32, 313.23)	1.38 (0.44,4.28)	1.70 (0.51,5.69)	0.56 (0.16,2.04)
Q4 (313.24, 620.83)	2.52 (0.51,12.42)	1.58 (0.16,15.78)	0.73 (0.07,8.10)
TyG-BMI(Per 1 unit increase)	1.01 (0.99,1.03)	1.00 (0.98,1.03)	1.02 (0.99,1.04)
TyG-WC			
Q1 (453.12, 792.26)	Reference	Reference	Reference
Q2 (792.27, 902.24)	1.53 (0.65,3.58)	1.27 (0.23,6.88)	0.88 (0.25,3.18)
Q3 (902.25, 1025.15)	1.80 (0.63,5.10)	3.64 (0.78,17.02)	1.17 (0.37,3.77)
Q4 (1025.16, 1648.81)	3.12 (0.79,12.32)	6.63 (0.39,112.03)	3.26 (1.03,10.34)*
TyG-WC(Per 1 unit increase)	1.00 (1.00,1.01)	1.00 (1.00,1.01)	1.00 (1.00,1.01)**
TyG-WHtR			
Q1 (2.63, 4.73)	Reference	Reference	Reference
Q2 (4.74, 5.40)	0.42 (0.16,1.05)	1.71 (0.37,7.85)	0.97 (0.28,3.31)
Q3 (5.41, 6.14)	1.54 (0.58,4.11)	1.29 (0.23,7.08)	1.11 (0.37,3.35)
Q4 (6.15, 10.23)	1.26 (0.27,5.83)	4.70 (0.89,24.88)	3.40 (1.00,11.63)
TyG-WHtR(Per 1 unit increase)	1.16 (0.65,2.06)	1.27 (0.54,3.00)	2.07 (1.22,3.51)**
TyG-WWI			
Q1 (60.85, 90.83)	Reference	Reference	Reference
Q2 (90.84, 98.38)	0.34 (0.11,1.02)	2.59 (0.13,51.77)	2.14 (0.82,5.58)
Q3 (98.39, 106.25)	0.58 (0.24,1.44)	1.58 (0.06,42.74)	2.92 (0.90,9.51)
Q4 (106.26, 144.15)	0.91 (0.37,2.25)	2.89 (0.08,109.86)	2.43 (0.93,6.31)
TyG-WWI(Per 1 unit increase)	1.01 (0.97,1.04)	1.01 (0.97,1.06)	1.04 (1.01,1.07)**
ABSI			
Q1 (0.60, 0.78)	Reference	Reference	Reference
Q2 (0.79, 0.81)	1.08 (0.27,4.24)	1.16 (0.17,8.10)	1.68 (0.35,8.10)
Q3 (0.82, 0.85)	1.56 (0.43,5.59)	3.19 (0.51,19.76)	3.05 (0.95,8.86)
Q4 (0.86, 0.99)	1.31 (0.38,4.51)	2.13 (0.23,19.98)	2.88 (0.86,9.64)
ABSI(Per 1 unit increase)	1.75 (0.04,77.20)	49.3 (6.19, 392)***	27.83 (0.89, 49.66)
LAP			
Q1 (3.07, 149.93)	Reference	Reference	Reference
Q2 (149.94, 202.77)	0.72 (0.31,1.64)	0.57 (0.15,2.16)	1.11 (0.32,3.89)
Q3 (202.78, 268.45)	1.41 (0.65,3.06)	0.59 (0.14,2.40)	0.98 (0.33,2.90)
Q4 (268.46, 604.96)	1.23 (0.54,2.80)	0.40 (0.06,2.78)	1.33 (0.35,5.10)
LAP(Per 1 unit increase)	1.00 (1.00,1.01)	1.00 (0.98,1.01)	1.00(0.99, 1.00)
VAI			
Q1 (0.15, 1.01)	Reference	Reference	Reference
Q2 (1.02, 1.68)	0.63 (0.26,1.53)	1.17 (0.15,8.93)	2.32 (0.80,6.74)
Q3 (1.69, 2.75)	0.84 (0.37,1.94)	4.64 (0.49,43.50)	2.66 (0.81,8.69)
Q4 (2.76, 11.39)	1.58 (0.66,3.80)	3.47 (0.27,44.20)	1.71 (0.50,5.83)
VAI(Per 1 unit increase)	1.13 (1.00,1.28)	1.22 (0.90,1.65)	1.23 (0.94,1.59)

*P < 0.05; **P < 0.01.

Multiple Cox regression model 3: Adjusted for Age, Sex, Education, Marital, PIR, Sedentary, Drinking status, Hyperlipidemia, CVD.

### Gender differences in analysis

Given that VAI and LAP were calculated based on gender, we further explored their predictive capacity for mortality through subgroup analysis in diabetes/prediabetes patients. The Cox regression analysis demonstrated a significant association between VAI and cardiovascular mortality specifically among males. Notably, after adjustment in Model 3, individuals in the Q3 (1.69, 2.75) and Q4 (2.76, 11.39) groups showed significantly increased cardiovascular mortality risks, with HR of 3.70 (95% CI: 1.21-11.3) and 3.43 (95% CI: 1.36-8.65), respectively. Furthermore, the analysis of ungrouped continuous variables indicated that for each one-unit increase in VAI, the cardiovascular mortality risk increased by 1.29 times. Finally, neither VAI nor LAP demonstrated significant differences in cancer-related mortality, with both showing no statistical significance (**Table 5**).

**Table 5:** Subgroup analysis of mortality risk based on gender.

All-cause mortality	Model 1 HR (95% CI)	Model 2 HR (95% CI)	Model3 HR (95% CI)
VAI (Female)			
Q1 (0.15, 1.02)	Reference	Reference	Reference
Q2 (1.02, 1.69)	1.26 (0.84, 1.88)	1.22 (0.80, 1.86)	1.19 (0.80, 1.77)
Q3 (1.69, 2.75)	1.22 (0.82, 1.83)	1.13 (0.75, 1.69)	1.07 (0.72, 1.58)
Q4 (2.76, 11.39)	1.33 (0.93, 1.92)	1.23 (0.84, 1.80)	1.14 (0.79, 1.65)
VAI (Per 1 unit increase)	1.07 (1.00, 1.15)*	1.06 (0.98, 1.14)	1.06 (0.98, 1.14)*
VAI (Male)			
Q1 (0.15, 1.02)	Reference	Reference	Reference
Q2 (1.02, 1.69)	0.78 (0.45, 1.33)	0.72 (0.42, 1.22)	0.79 (0.45, 1.38)
Q3 (1.69, 2.75)	1.27 (0.77, 2.09)	1.26 (0.73, 2.21)	1.44 (0.81, 2.57)
Q4 (2.76, 11.39)	1.37 (0.83, 2.27)	1.39 (0.85, 2.28)	1.69 (0.99, 2.88)
VAI (Per 1 unit increase)	1.10 (0.98, 1.23)	1.12 (1.00, 1.25)*	1.16 (1.04, 1.29)**
LAP (Female)			
Q1 (3.07, 149.94)	Reference	Reference	Reference
Q2 (149.94, 202.78)	0.83 (0.60, 1.14)	0.92 (0.61, 1.38)	0.96 (0.66, 1.40)
Q3 (202.78, 268.45)	0.78 (0.56, 1.07)	0.92 (0.61, 1.37)	0.94 (0.63, 1.42)
Q4 (268.46, 604.96)	0.85 (0.63, 1.13)	0.88 (0.58, 1.34)	0.91 (0.60, 1.38)
LAP (Per 1 unit increase)	1.00 (1.00, 1.00)	1.00 (1.00, 1.00)	1.00 (1.00, 1.00)
LAP (Male)			
Q1 (3.07, 149.94)	Reference	Reference	Reference
Q2 (149.94, 202.78)	0.68 (0.45, 1.01)	0.72 (0.44, 1.18)	0.77 (0.46, 1.28)
Q3 (202.78, 268.45)	0.88 (0.61, 1.27)	0.95 (0.55, 1.61)	1.03 (0.60, 1.78)
Q4 (268.46, 604.96)	1.26 (0.71, 2.23)	1.63 (0.55, 4.77)	1.75 (0.58, 5.25)
LAP (Per 1 unit increase)	1.00 (1.00, 1.00)	1.00 (1.00, 1.01)	1.00 (1.00, 1.01)
Cardiovascular mortality	Model 1 HR (95% CI)	Model 2 HR (95% CI)	Model3 HR (95% CI)
VAI (Female)			
Q1 (0.15, 1.02)	Reference	Reference	Reference
Q2 (1.02, 1.69)	1.08 (0.59, 1.98)	1.02 (0.54, 1.94)	0.94 (0.50, 1.77)
Q3 (1.69, 2.75)	0.93 (0.47, 1.83)	0.87 (0.41, 1.83)	0.84 (0.41, 1.71)
Q4 (2.76, 11.39)	1.03 (0.53, 2.00)	0.92 (0.47, 1.80)	0.81 (0.43, 1.55)
VAI (Per 1 unit increase)	1.01 (0.87, 1.18)	0.99 (0.85, 1.16)	0.98 (0.84, 1.14)
VAI (Male)			
Q1 (0.15, 1.02)	Reference	Reference	Reference
Q2 (1.02, 1.69)	1.59 (0.66, 3.78)	1.40 (0.52, 3.74)	1.55 (0.59, 4.07)
Q3 (1.69, 2.75)	3.51 (1.34, 9.25)*	3.71 (1.27, 10.9)*	3.70 (1.21, 11.3)*
Q4 (2.76, 11.39)	3.03 (1.28, 7.13)*	3.08 (1.20, 7.90)*	3.43 (1.36, 8.65)**
VAI (Per 1 unit increase)	1.26 (1.07, 1.49)**	1.29 (1.08, 1.55)**	1.29 (1.09, 1.52)**
LAP (Female)			
Q1 (3.07, 149.94)	Reference	Reference	Reference
Q2 (149.94, 202.78)	0.62 (0.33, 1.15)	0.70 (0.34, 1.44)	0.77 (0.37, 1.60)
Q3 (202.78, 268.45)	0.57 (0.33, 1.00)*	0.63 (0.33, 1.18)	0.67 (0.35, 1.31)
Q4 (268.46, 604.96)	0.72 (0.44, 1.20)	0.66 (0.30, 1.46)	0.75 (0.32, 1.76)
LAP (Per 1 unit increase)	1.00 (1.00, 1.00)	1.00 (0.99, 1.00)	1.00 (1.00, 1.00)
LAP (Male)			
Q1 (3.07, 149.94)	Reference	Reference	Reference
Q2 (149.94, 202.78)	0.35 (0.17, 0.76)**	0.33 (0.12, 0.88)*	0.36 (0.13, 0.94)*
Q3 (202.78, 268.45)	1.11 (0.62, 2.00)	0.90 (0.30, 2.70)	0.94 (0.31, 2.82)
Q4 (268.46, 604.96)	1.74 (0.65, 4.63)	1.32 (0.11, 16.4)	1.36 (0.11, 16.6)
LAP (Per 1 unit increase)	1.00 (1.00, 1.01)	1.00 (1.00, 1.01)	1.00 (1.00, 1.01)
Cancer mortality	Model 1 HR (95% CI)	Model 2 HR (95% CI)	Model 3 HR (95% CI)
VAI (Female)			
Q1 (0.15, 1.02)	Reference	Reference	Reference
Q2 (1.02, 1.69)	0.89 (0.32,2.53)	0.78 (0.26,2.33)	0.76 (0.26,2.21)
Q3 (1.69, 2.75)	1.30 (0.43,3.97)	1.29 (0.43,3.94)	1.21 (0.39,3.73)
Q4 (2.76, 11.39)	1.71 (0.66,4.42)	1.58 (0.61,4.15)	1.45 (0.53,3.96)
VAI (Per 1 unit increase)	1.19 (1.06,1.34)**	1.17 (1.04,1.32)*	1.16 (0.93,1.31)
VAI (Male)			
Q1 (0.15, 1.02)	Reference	Reference	Reference
Q2 (1.02, 1.69)	1.00 (0.39,2.55)	0.94 (0.34,2.64)	1.21 (0.43,3.43)
Q3 (1.69, 2.75)	1.12 (0.46,2.72)	1.05 (0.41,2.69)	1.41 (0.59,3.37)
Q4 (2.76, 11.39)	1.83 (0.68,4.91)	1.91 (0.58,6.26)	2.81 (0.88,8.93)
VAI (Per 1 unit increase)	1.07 (0.88,1.29)	1.06 (0.86,1.31)	1.12 (0.93,1.33)
LAP (Female)			
Q1 (3.07, 149.94)	Reference	Reference	Reference
Q2 (149.94, 202.78)	0.48 (0.19,1.19)	0.57 (0.22,1.50)	0.57 (0.22,1.45)
Q3 (202.78, 268.45)	0.99 (0.42,2.33)	1.18 (0.49,2.84)	1.09 (0.46,2.58)
Q4 (268.46, 604.96)	0.94 (0.41,2.18)	1.11 (0.47,2.61)	0.99 (0.42,2.34)
LAP (Per 1 unit increase)	1.00 (1.00,1.00)	1.00 (1.00,1.01)	1.00 (1.00,1.01)
LAP (Male)			
Q1 (3.07, 149.94)	Reference	Reference	Reference
Q2 (149.94, 202.78)	0.65 (0.26,1.62)	0.66 (0.26,1.73)	0.67 (0.25,1.76)
Q3 (202.78, 268.45)	0.80 (0.35,1.83)	0.88 (0.30,2.56)	0.92 (0.31,2.69)
Q4 (268.46, 604.96)	0.76 (0.26,2.22)	0.95 (0.29,3.16)	1.03 (0.32,3.36)
LAP (Per 1 unit increase)	1.00 (0.99,1.00)	1.00 (0.99,1.01)	1.00 (0.99,1.01)

*P < 0.05; **P < 0.01.

Multiple Cox regression model: Model 1: Adjusted for Age, Race; Model 2: Adjusted for Age, Race, Education, Marital, PIR, Sedentary, Drinking status; Model 3: Adjusted for Age, Race, Education, Marital, PIR, Sedentary, Drinking status, Hyperlipidemia, CVD.

LAP, Lipid accumulation product; VAI, Visceral adiposity index.

### Trend analysis of obesity and lipid-related indices with mortality

**Figure 4:**
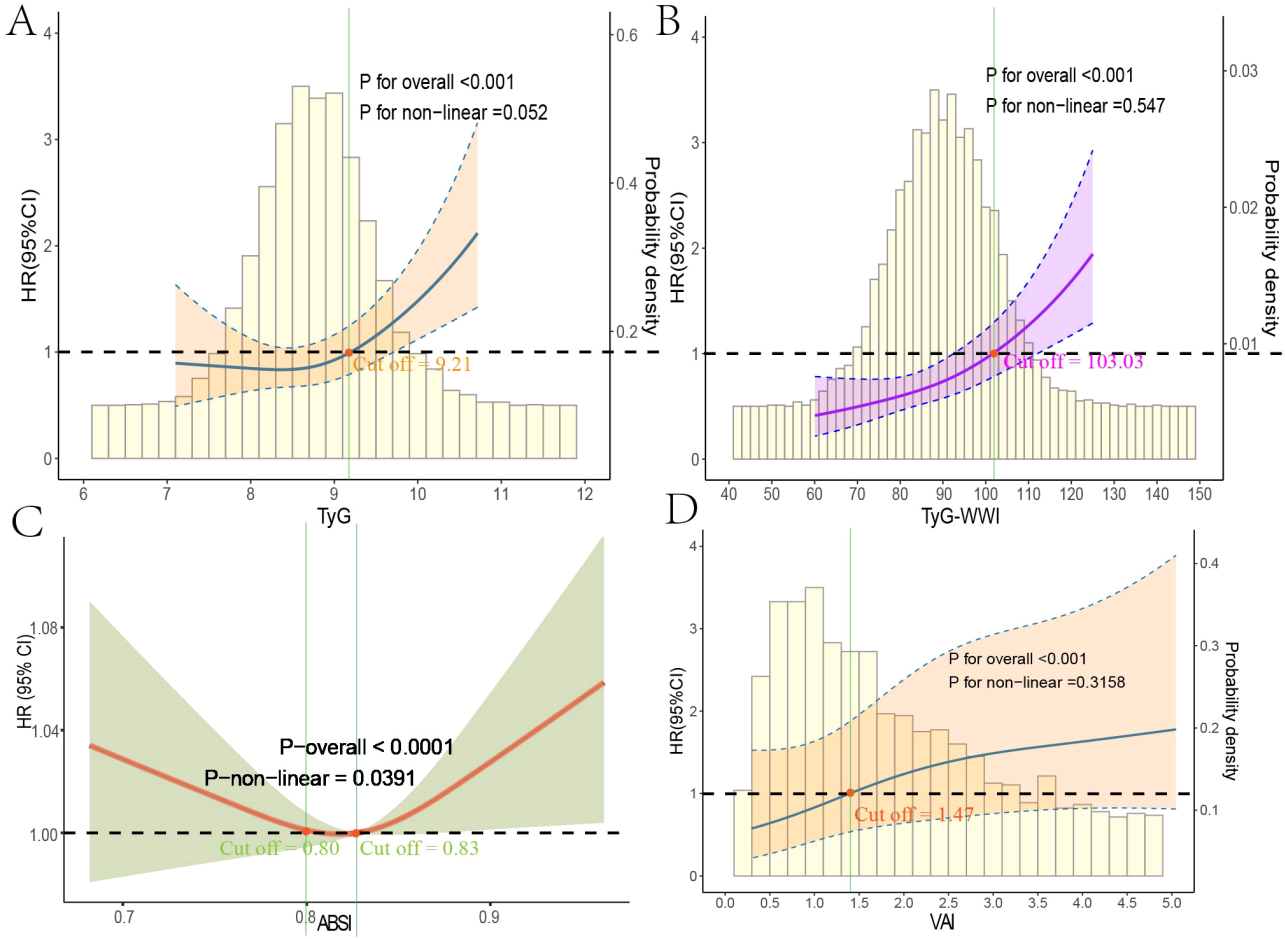
Restricted cubic spline regression analysis of obesity and lipid-related indices with mortality. This figure presents spline analysis of mortality risk for obesity and lipid-related indices, accompanied by the background frequency distribution histogram. Solid lines represent the hazard ratios (HR) adjusted for multivariable covariates (Age, Gender, Race, Education, Marital Status, Poverty Income Ratio (PIR), Sedentary Behavior, Drinking Status, Hyperlipidemia, and Cardiovascular disease (CVD)). The shaded areas indicate the 95% confidence intervals derived from the RCS regression. TyG **(A)** and TyG-WWI **(B)** show linear relationships with all-cause mortality, while ABSI **(C)** indicates a non-linear relationship. VAI **(D)** demonstrates a linear relationship with cardiovascular mortality in male patients, where all red points correspond to cutoff values for HR > 1.

Using multivariable-adjusted RCS analysis, we visualized the associations of various indices with all-cause and cardiovascular mortality in diabetes/prediabetes patients. The analysis of all-cause mortality revealed that both TyG and TyG-WWI displayed a linear relationship with all-cause mortality (overall P-values < 0.0001), with cutoff points where the HR exceeded 1 at 9.21 and 103.03, respectively (**Figures 4A, B**). In contrast, ABSI exhibited a non-linear relationship with all-cause mortality, with a non-linear P-value of 0.0391, showing cutoff points at 0.80 and 0.83 (**Figure 4C**). Additionally, among male diabetes/prediabetes patients, moderate to high levels of VAI (Q3: 1.69, 2.75; Q4: 2.76, 11.39) were associated with a clear linear relationship to cardiovascular mortality (overall P-value < 0.0001), with a cutoff point where HR exceeded 1 at 1.47 (**Figure 4D**).

### Evaluation of machine learning models

**Figure 5:**
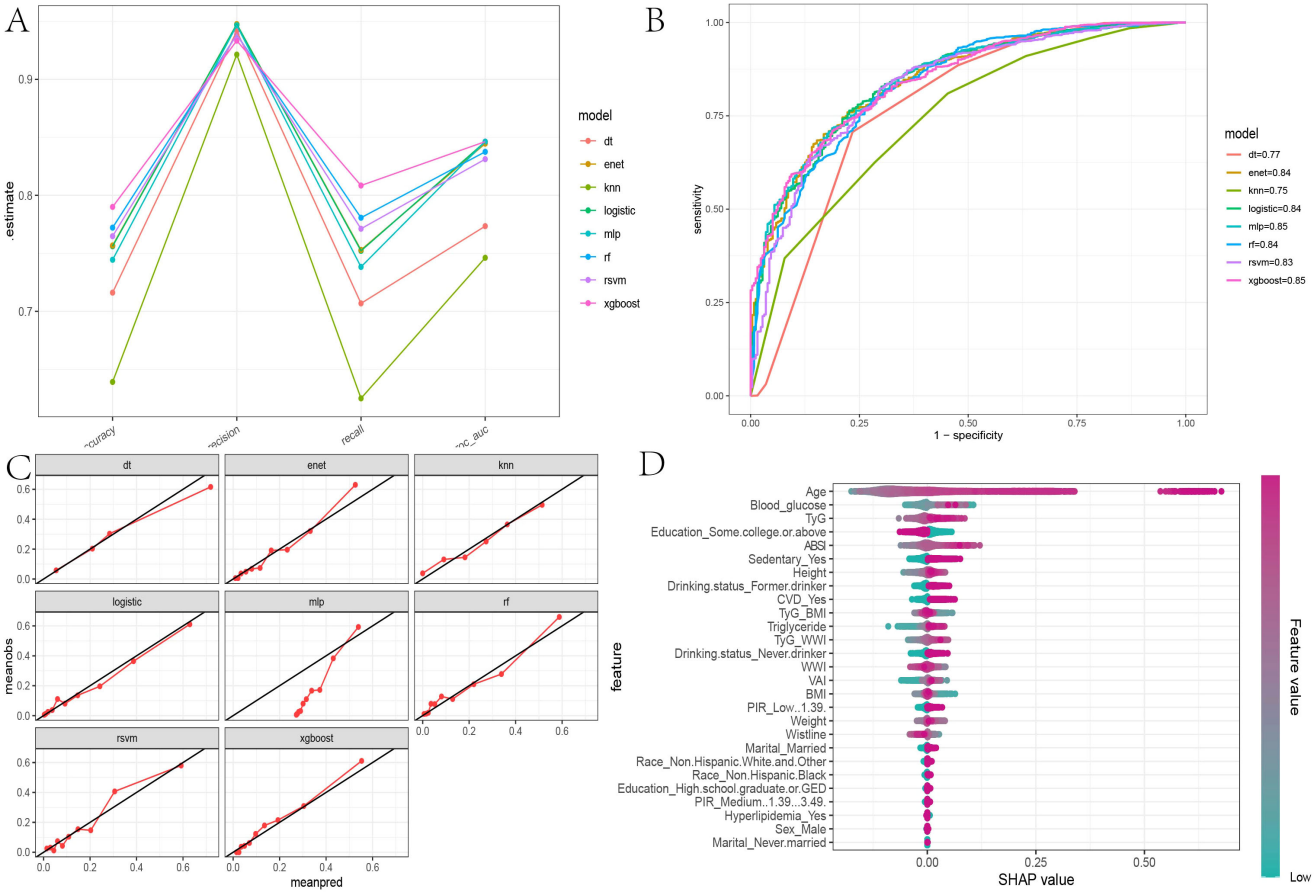
Evaluation of machine learning models and SHAP beeswarm Plot. **(A)** The parallel coordinate plot assesses the efficacy of eight machine learning algorithms based on accuracy, precision, recall, and ROC AUC calculations found in **Supplementary Table S2**; **(B)** Comparison of eight machine learning algorithms on ROC curves; **(C)** Calibration curves for eight machine learning algorithms; **(D)** SHAP interpretability beeswarm plot incorporating all obesity and lipid-related indices associated with mortality risk, illustrating the cumulative impact of each feature on mortality risk and sorted by importance.

After determining that TyG, TyG-WWI, and ABSI are significant risk factors for all-cause mortality in diabetes/prediabetes patients, we aimed to assess the importance of these indices and their contributions to mortality risk by comparing eight ML algorithms: XGBoost, DT, SVM, Enet, MLP, RF, and KNN. The results indicated that the XGBoost model achieved the highest area under the curve (AUC) value of 0.85, with an accuracy of 0.79, precision of 0.94, and recall of 0.81, indicating strong performance across various metrics (**Figures 5A, B**, **Supplementary Table S5**). Furthermore, the calibration curve showed good consistency between the predicted probabilities from the XGBoost model and the actual probabilities (**Figure 5C**). Thus, we selected XGBoost as the optimal ML model for predicting mortality risk in this study. Subsequently, we used the SHAP model to interpret and visualize feature importance. The beeswarm plot illustrates the cumulative impact of each feature on mortality risk, arranged in descending order of importance. Positive SHAP values indicate that increasing feature values are directly correlated with higher mortality risk, with larger SHAP values contributing more significantly to the predictions.

**Figure 6:**
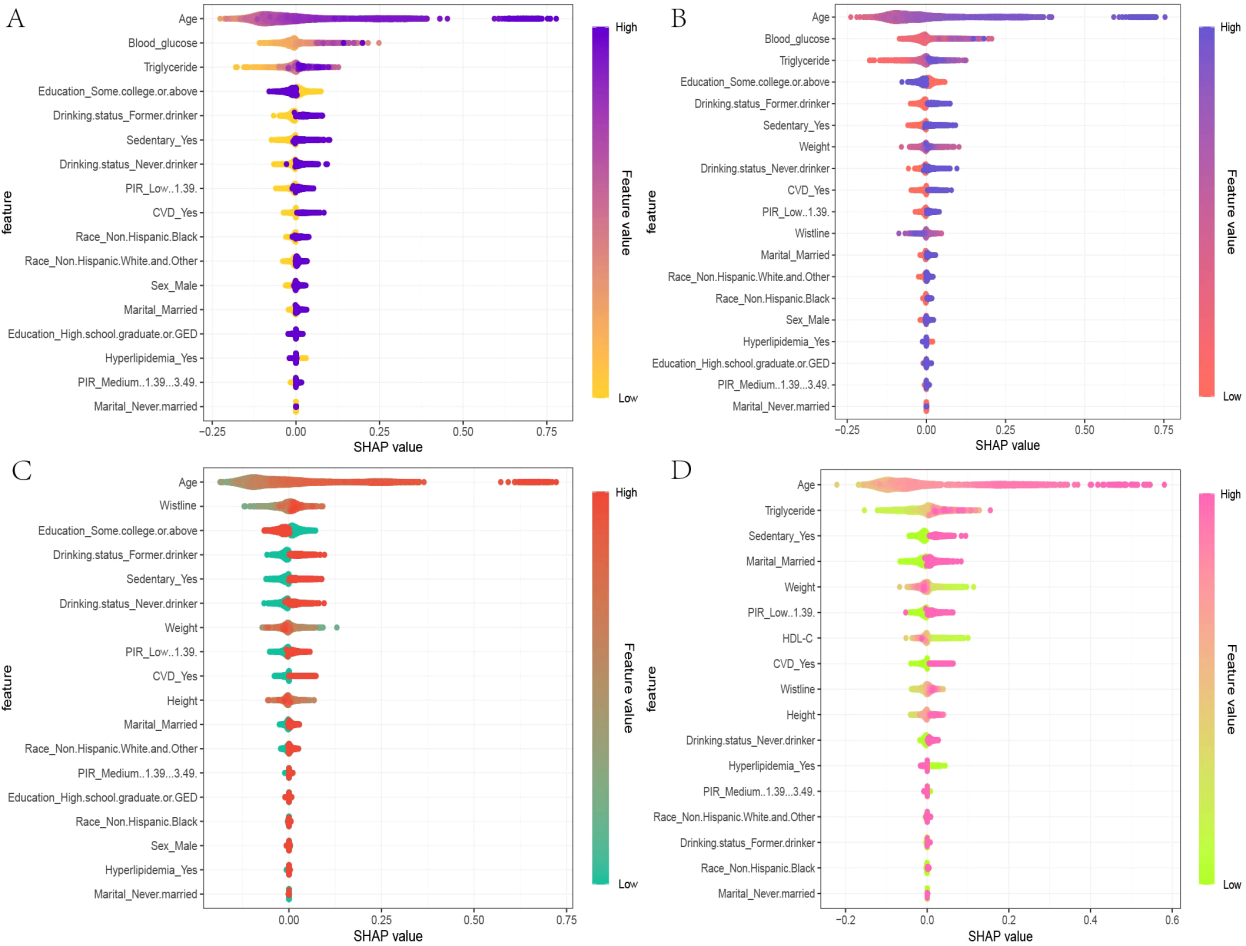
SHAP beeswarm plots for the four indices in the optimal XGBoost model. **(A)** The SHAP beeswarm plot for TyG, **(B)** TyG-WWI, **(C)** ABSI, and **(D)** VAI models provides a global interpretation of how each component of these indices predicts the risk of mortality in patients with diabetes/prediabetes. The beeswarm plots illustrate the cumulative impact of each feature on mortality risk, arranged in descending order of importance. In the plots, the Feature values are represented using a gradient color scheme that indicates the magnitude of each feature variable. Positive SHAP values imply that the feature values are positively correlated with an increased risk of mortality, with larger SHAP values contributing more significantly to mortality predictions. This visual representation helps in understanding how each component of TyG, TyG-WWI, ABSI, and VAI influences the overall risk of death among individuals diagnosed with diabetes or prediabetes.

In the global interpretability of the optimal XGBoost model, age emerged as the most significant contributor to mortality risk, consistent with clinical expectations. In the overall model that included covariates, the importance ranking of obesity and lipid-related indices was as follows: TyG > ABSI > TyG-WWI (**Figure 5D**). In the TyG-related model, apart from age, fasting blood glucose and triglycerides ranked second and third, respectively. Notably, very low fasting blood glucose also increased mortality risk (**Figure 6A**). A similar trend was observed in the TyG-WWI model, with fasting blood glucose and triglycerides carrying substantial weight, followed by weight and waist circumference (**Figure 6B**). In the ABSI model, waist circumference was the most significant factor, followed by weight and height, with very low weight significantly increasing the risk of mortality (**Figure 6C**).

In summary, both blood glucose-related indices and anthropometric measures substantially influence mortality risk in patients with diabetes/prediabetes. For cardiovascular mortality risk in male patients, the feature importance for VAI was ranked as follows: triglycerides, HDL-C, height, waist circumference, and weight, with triglycerides, waist circumference, and weight positively contributing to mortality risk, while other indicators showed a negative impact (**Figure 6D**).

## Discussion

This cross-sectional study systematically explores the relationship between obesity and lipid-related indices and all-cause and cardiovascular mortality in patients with diabetes/prediabetes in the United States. Additionally, we employed machine learning methods to assess and compare the predictive capabilities of these indices regarding mortality risk. The findings indicate that TyG > 8.75, ABSI > 0.82, and TyG-WWI > 98.39 are positively correlated with all-cause mortality in diabetes/prediabetes patients, with TyG > 8.75 also showing a significant positive correlation with cardiovascular mortality. We observed a non-linear trend in the relationships between ABSI and TyG-WWI and all-cause mortality, while TyG exhibited a significant linear correlation with both all-cause and cardiovascular mortality.

Among the machine learning algorithms, the XGBoost model demonstrated the best predictive performance, and the SHAP analysis revealed that TyG is the most significant contributor to all-cause mortality in patients with diabetes/prediabetes. In addition to age, high levels of fasting blood glucose and triglycerides were significant contributors. The remaining indices were ranked in terms of their contribution as follows: ABSI, TyG-BMI, and TyG-WWI. In the subgroup analysis based on gender, we found that moderate to high levels of VAI (>1.69) were positively correlated with cardiovascular mortality in male patients with diabetes and prediabetes, displaying a linear relationship, with triglycerides being the most significant contributor.

We recognize that TyG is an important indicator of IR, a core issue in diabetes/prediabetes. The significance of TyG in predicting risk for patients with diabetes and prediabetes should not be overlooked. This study also highlights, for the first time that the TyG-related index, TyG-WWI, can effectively predict all-cause mortality risk in diabetes and prediabetes patients, and TyG can also be used to predict cardiovascular mortality risk. The effectiveness of TyG in assessing insulin resistance is partly attributed to its strong sensitivity and specificity, as well as its broad clinical applicability (17). The association between TyG and mortality in diabetes patients, along with poor cardiovascular outcomes, may be influenced by various factors. First, insulin resistance leads to dysregulation of glucose and lipid metabolism, exacerbating inflammation and oxidative stress in the body, which accelerates biological aging (23) and promotes the development of atherosclerosis and CHD (24). Second, insulin resistance can elevate reactive oxygen species (ROS) levels, damaging vascular endothelium (25), leading to excessive platelet activation and potentially triggering thrombosis (26). This series of issues further contributes to cardiovascular diseases, which are among the leading causes of mortality. Therefore, this not only explains why TyG is used to predict the incidence of cardiovascular diseases (27) but also clarifies why TyG is more closely associated with cardiovascular mortality risk than other indices in this study (28).

In this study, we found that the average BMI of all participants was 31 kg/m², indicating a significant obesity risk among patients with diabetes and prediabetes in the United States. It is important to consider that BMI may not effectively distinguish between muscle and fat composition; typically, higher fat content is associated with lower life expectancy, while higher muscle mass may contribute to increased longevity (29). The relationship between BMI and mortality risk is complex, with meta-analyses suggesting a U-shaped non-linear association (30). This phenomenon partially explains the paradox whereby individuals with a high BMI may have a longer lifespan than those with a lower BMI in populations with diabetes (31). In our study, we also conducted subgroup analyses based on BMI levels and found that obesity exhibited more dangerous tendencies across several key indicators including blood glucose, insulin, and TyG index. Interestingly, we also observed that BMI levels in the mortality group were slightly lower than those in the survival group; however, the proportions of hyperlipidemia and CVD were higher. This finding suggests that, in addition to TyG, it is essential to consider other obesity-related indices in our analyses. The results also showed that TyG-WWI plays a significant role in predicting all-cause mortality risk. The WWI standardizes waist circumference relative to body weight, emphasizing abdominal obesity while minimizing the association with BMI. Recent studies have recognized WWI as superior to BMI in predicting diabetes (32). Our research further integrates WWI with TyG to enhance predictive ability regarding mortality risk in patients with diabetes/prediabetes.

Additionally, ABSI, a newly developed body shape index based on waist circumference, weight, and height, is positively associated with visceral fat accumulation (33). Visceral fat accumulation is linked to various adverse outcomes due to excess fatty acid buildup (34), increased triglyceride synthesis and secretion (35), and lower levels of protective factors (PPAR-γ, glycogen synthase, and leptin) (36). ABSI has been validated as an independent predictor of survival rates (33). A 20-year follow-up study by Tate J demonstrated a linear positive correlation between ABSI and all-cause mortality in diabetes patients (37). A notable advantage of ABSI over TyG is its measurement is non-invasive, allowing patients with diabetes/prediabetes to conveniently track changes in this index.

In our gender-based analysis, we found that VAI was associated with cardiovascular mortality risk. Previous research, including that by Marco C, identified VAI as an important indicator of visceral fat function and insulin sensitivity (38). In our study, an association between VAI and cardiovascular mortality risk was observed only in males, similar with findings from Shi Y regarding gender differences affecting VAI (39). The impact of VAI regarding gender differences may stem from variations in insulin sensitivity and differences in body fat distribution due to hormonal levels. Relevant data indicate that, at a given body type, women usually have approximately 10% higher body fat percentages than men (40), and women’s body fat percentages have consistently been higher throughout life (41). This may suggest that men are more sensitive to the health implications of visceral fat accumulation. Therefore, findings regarding gender differences in our study warrant further exploration, potentially guiding future research directions.

Indicators such as TyG, TyG-WWI, ABSI, and VAI effectively reflect individual body composition and lipid profiles in the blood. Based on our findings, we recommend weight loss interventions be prioritized in improving health outcomes for patients with diabetes and prediabetes. However, these strategies should emphasize reducing visceral fat rather than focusing solely on overall weight loss. We suggest implementing effective exercise modalities, including resistance training, aerobic exercise, and overall conditioning. The benefits of these exercise types are reflected in some indicators from our study; for instance, reducing WC can effectively lower ABSI and VAI, suggesting potential improvements in patients’ life expectancy, even without significant changes in body weight.

Naturally, this study has several unresolved issues. Firstly, due to the limitations of cross-sectional study designs, we cannot establish clear causal relationships distinguishing TyG, TyG-WWI, ABSI, VAI, and their associations with mortality risk in patients with diabetes/prediabetes. However, the NHANES dataset’s bolsters lies in its large and nationally representative sample, which enhances the statistical power of our analyses and bolsters the reliability of our findings. Secondly, the study population comprised individuals exclusively from the United States, limiting the generalizability of our conclusions. Responses to obesity and related metabolic indicators may differ significantly across regions, cultural backgrounds, or dietary habits. Therefore, our results may not apply to other populations, and future research should consider broader demographic studies to validate the universality and applicability of these findings.

## Conclusion

This study represents the first comprehensive assessment of the associations between obesity, lipid-related indices, and all-cause and cardiovascular mortality risk in patients with diabetes/prediabetes. The results indicate that TyG is closely related to all-cause and cardiovascular mortality in patients with diabetes/prediabetes and demonstrates superior predictive capability compared to other indices. This finding underscores the potential role of TyG as an effective biomarker in the clinical management of diabetes/prediabetes. Furthermore, TyG-WWI and ABSI also effectively predict all-cause mortality risk, while VAI shows a significant association with cardiovascular mortality specifically in male patients. These indicators assess life expectancy from multiple dimensions and suggest a greater focus on the rationality of body fat distribution rather than solely on BMI or overall weight changes. This shift in focus can help optimize long-term management and intervention strategies for patients with diabetes and prediabetes.

## Data Availability

The NHANES data in this study is sourced from the Centers for Disease Control and Prevention, and all data is freely accessible at: https://wwwn.cdc.gov/Nchs/Nhanes/.
