# Supplementary material for: The association of obesity and lipid-related indicators with all-cause and cardiovascular mortality risks in patients with diabetes or prediabetes: a cross-sectional study based on machine learning algorithms
Source: Front Endocrinol (Lausanne). 2025 Jun 2;16:1492082. doi: 10.3389/fendo.2025.1492082 (PMC12171389; doi:10.3389/fendo.2025.1492082)
Supplement: Supplementary file 1 [file Table1.docx]

Supplementary materials - Catalogue

[1. Table S1. Characteristics of participants according to Cardiovascular mortality. (NHANES 1999-2018, N = 3,615) 2](#_Toc193841249)

[2. Table S2. All-cause mortality HR based on quartiles of obesity and lipid-related indices 4](#_Toc193841250)

[3. Table S3. Cardiovascular mortality HR based on quartiles of obesity and lipid-related indices 6](#_Toc193841251)

[4. Table S4. Cancer mortality HR based on quartiles of obesity and lipid-related indices 8](#_Toc193841252)

[5. Table S5. Indicators for Evaluating the Performance of Machine Learning Models 10](#_Toc193841253)

[6. Table S6. Characteristics of participants according to BMI. 11](#_Toc193841254)

# Table S1. Characteristics of participants according to Cardiovascular mortality. (NHANES 1999-2018, N = 3,615)

| **Characteristic** | **Overall**, N = 3615 (100%)^1,2^ | **Survival Group**, N = 3418 (96%)^1,2^ | **Mortality Group**, N = 197 (4.0%)^1,2^ | **P Value** |
| --- | --- | --- | --- | --- |
| **Age (years)** | 53.2 (16.1) | 52.4 (15.8) | 70.7 (11.7) | **<0.001** |
| **Sex** |  |  |  | 0.5 |
| *Female* | 2,193 (58%) | 2,078 (58%) | 115 (61%) |  |
| *Male* | 1,422 (42%) | 1,340 (42%) | 82 (39%) |  |
| **Race** |  |  |  | **0.002** |
| *Non-Hispanic White and Other* | 1,765 (72%) | 1,644 (71%) | 121 (79%) |  |
| *Hispanic* | 1,001 (16%) | 967 (16%) | 34 (6.7%) |  |
| *Non-Hispanic Black* | 849 (13%) | 807 (13%) | 42 (14%) |  |
| **Marital** |  |  |  | **0.049** |
| *Divorced* | 2,014 (59%) | 1,923 (60%) | 91 (49%) |  |
| *Married* | 1,464 (38%) | 1,365 (38%) | 99 (48%) |  |
| *Never married* | 137 (2.6%) | 130 (2.6%) | 7 (3.0%) |  |
| **PIR** |  |  |  | **0.020** |
| *High(>3.49)* | 968 (38%) | 932 (38%) | 36 (25%) |  |
| *Low(≤1.39)* | 1,267 (25%) | 1,184 (25%) | 83 (34%) |  |
| *Medium(>1.39,<=3.49)* | 1,380 (37%) | 1,302 (37%) | 78 (41%) |  |
| **Drinking.status** |  |  |  | **<0.001** |
| *Current drinker* | 1,448 (47%) | 1,411 (48%) | 37 (25%) |  |
| *Former drinker* | 946 (24%) | 867 (23%) | 79 (38%) |  |
| *Never-drinker* | 1,221 (29%) | 1,140 (28%) | 81 (37%) |  |
| **Hyperlipidemia** | 2,900 (80%) | 2,729 (80%) | 171 (87%) | 0.053 |
| **CVD** | 498 (12%) | 421 (10%) | 77 (39%) | **<0.001** |
| **Weight** | 87 (23) | 87 (23) | 80 (24) | **0.007** |
| **Height** | 167 (10) | 167 (10) | 164 (10) | **<0.001** |
| **Waist circumference** | 104 (17) | 104 (17) | 103 (17) | 0.4 |
| **Insulin** | 16 (18) | 16 (18) | 15 (16) | 0.5 |
| **Triglyceride** | 126 (67) | 125 (66) | 152 (74) | **<0.001** |
| **Blood glucose** | 117 (36) | 117 (35) | 124 (42) | **0.034** |
| **WHtR** | 0.63 (0.10) | 0.63 (0.10) | 0.63 (0.10) | 0.8 |
| **WWI** | 11.27 (0.80) | 11.26 (0.80) | 11.62 (0.77) | **<0.001** |
| **BMI** | 31 (7) | 31 (7) | 30 (8) | 0.067 |
| **TyG-BMI** | 274 (72) | 274 (72) | 268 (75) | 0.5 |
| **TyG-WHtR** | 5.50 (1.05) | 5.50 (1.05) | 5.68 (1.01) | 0.068 |
| **TyG-WWI** | 99 (11) | 98 (11) | 105 (10) | **<0.001** |
| **TyG-WC** | 915 (175) | 915 (175) | 929 (173) | 0.4 |
| **TyG** | 8.74 (0.62) | 8.73 (0.62) | 9.00 (0.61) | **<0.001** |
| **ABSI** | 0.82 (0.05) | 0.82 (0.05) | 0.85 (0.05) | **<0.001** |
| **LAP** | 215 (92) | 215 (92) | 214 (87) | 0.9 |
| **VAI** | 2.10 (1.50) | 2.08 (1.49) | 2.50 (1.69) | **0.007** |

1Mean ± SD for continuous; n (%) for categorical

2t-test adapted to complex survey samples; chi-squared test with Rao & Scott's second-order correction.

TyG: Triglyceride Glucose; TyG-BMI: Triglyceride Glucose - Body Mass Index; TyG-WHtR: Triglyceride Glucose - Waist to Height Ratio; TyG-WWI: Triglyceride Glucose - Weight Adjusted Waist Index; TyG-WC: Triglyceride Glucose - Waist Circumference; ABSI: A Body Shape Index; LAP: Lipid Accumulation Product; VAI: Visceral Adiposity Index.

# Table S2. All-cause mortality HR based on quartiles of obesity and lipid-related indices

| **All-cause mortality** | **Model 1**  **HR (95% CI)** | **Model 2**  **HR (95% CI)** | **Model3**  **HR (95% CI)** |
| --- | --- | --- | --- |
| **TyG** |  |  |  |
| Q1 (6.56,8.33) | Reference | Reference | Reference |
| Q2 (8.34,8.74) | 1.17(0.87, 1.57) | 1.05(0.78, 1.42) | 1.07(0.80, 1.45) |
| Q3 (8.75,9.17) | 1.12(0.82, 1.53) | 0.98(0.71, 1.37) | 0.98(0.69, 1.38) |
| Q4 (9.18,11.03) | 1.62(1.21, 2.15)** | 1.44(1.07, 1.94)* | 1.49(1.09, 2.03)* |
| **TyG(Per 1 unit increase)** | 1.41(1.20, 1.67)*** | 1.35(1.13, 1.61)** | 1.40(1.16, 1.68)*** |
| **TyG-BMI** |  |  |  |
| Q1 (115.40, 223.37) | Reference | Reference | Reference |
| Q2 (223.38, 262.31) | 0.76(0.58, 0.99)* | 0.71(0.53, 0.97)* | 0.73(0.54, 1.08) |
| Q3 (262.32, 313.23) | 0.89(0.68, 1.18) | 0.87(0.65, 1.17) | 0.87(0.65, 1.16) |
| Q4 (313.24, 620.83) | 1.01(0.78, 1.31) | 0.86(0.66, 1.13) | 0.86(0.64, 1.14) |
| **TyG-BMI(Per 1 unit increase)** | 1.00(1.00, 1.00) | 1.00(1.00, 1.00) | 1.00(1.00, 1.00) |
| **TyG-WC** |  |  |  |
| Q1 (453.12, 792.26) | Reference | Reference | Reference |
| Q2 (792.27, 902.24) | 0.96(0.72, 1.29) | 0.94(0.69, 1.28) | 0.96(0.69, 1.33) |
| Q3 (902.25, 1025.15) | 0.93(0.70, 1.22) | 0.88(0.66, 1.19) | 0.88(0.64, 1.20) |
| Q4 (1025.16, 1648.81) | 1.29(0.96, 1.74) | 1.14(0.82, 1.57) | 1.13(0.80, 1.59) |
| **TyG-WC(Per 1 unit increase)** | 1.00(1.00, 1.00)* | 1.00(1.00, 1.00) | 1.00(1.00, 1.00) |
| **TyG-WHtR** |  |  |  |
| Q1 (2.63, 4.73) | Reference | Reference | Reference |
| Q2 (4.74, 5.40) | 0.84(0.61, 1.16) | 0.82(0.59, 1.15) | 0.86(0.60, 1.22) |
| Q3 (5.41, 6.14) | 1.07(0.78, 1.46) | 1.08(0.80, 1.44) | 1.03(0.73, 1.46) |
| Q4 (6.15, 10.23) | 1.22(0.92, 1.62) | 1.08(0.80, 1.44) | 1.08(0.78, 1.49) |
| **TyG-WHtR(Per 1 unit increase)** | 1.14(1.02, 1.27)* | 1.08(0.96, 1.21) | 1.09(0.96, 1.23) |
| **TyG-WWI** |  |  |  |
| Q1 (60.85, 90.83) | Reference | Reference | Reference |
| Q2 (90.84, 98.38) | 0.83(0.61, 1.14) | 0.78(0.55, 1.11) | 0.87(0.59, 1.30) |
| Q3 (98.39, 106.25) | 1.10(0.81, 1.49) | 1.04(0.75, 1.43) | 1.17(0.81, 1.71) |
| Q4 (106.26, 144.15) | 1.53(1.12, 2.11)** | 1.39(1.00, 1.95)* | 1.52(1.02, 2.26)* |
| **TyG-WWI(Per 1 unit increase)** | 1.02(1.01, 1.03)*** | 1.02(1.01, 1.03)*** | 1.02(1.01, 1.03)*** |
| **ABSI** |  |  |  |
| Q1 (0.60, 0.78) | Reference | Reference | Reference |
| Q2 (0.79, 0.81) | 1.09(0.76, 1.57) | 1.16(0.79, 1.68) | 1.27(0.87, 1.87) |
| Q3 (0.82, 0.85) | 1.56(1.11, 2.19)* | 1.72(1.21, 2.45)** | 1.80(1.23, 2.64)** |
| Q4 (0.86, 0.99) | 1.57(1.15, 2.13)** | 1.61(1.16, 2.23)** | 1.68(1.17, 2.41)** |
| **ABSI(Per 1 unit increase)** | 56.1(6.61, 476)*** | 49.3(6.19, 392)*** | 48.6(5.49, 430)*** |
| **LAP** |  |  |  |
| Q1 (3.07, 149.93) | Reference | Reference | Reference |
| Q2 (149.94, 202.77) | 0.78(0.60, 1.00) | 0.79(0.59, 1.06) | 0.83(0.63, 1.11) |
| Q3 (202.78, 268.45) | 0.82(0.64, 1.05) | 0.84(0.66, 1.08) | 0.88(0.68, 1.13) |
| Q4 (268.46, 604.96) | 0.96(0.74, 1.26) | 0.91(0.68, 1.21) | 0.95(0.70, 1.28) |
| **LAP(Per 1 unit increase)** | 1.00 (1.00, 1.00) | 1.00 (1.00, 1.00) | 1.00(1.00, 1.00) |
| **VAI** |  |  |  |
| Q1 (0.15, 1.01) | Reference | Reference | Reference |
| Q2 (1.02, 1.68) | 1.05(0.76, 1.45) | 0.99(0.71, 1.38) | 0.98(0.71, 1.37) |
| Q3 (1.69, 2.75) | 1.21(0.88, 1.67) | 1.10(0.79, 1.54) | 1.08(0.76, 1.52) |
| Q4 (2.76, 11.39) | 1.34(1.01, 1.77)* | 1.22(0.91, 1.63) | 1.24(0.91, 1.67) |
| **VAI(Per 1 unit increase)** | 1.08(1.02, 1.15)** | 1.06(1.00, 1.13)* | 1.07(1.01, 1.14)* |

*P < 0.05; **P < 0.01; ***P < 0.001.

Multiple Cox regression model: Model 1: Adjusted for Age, Gender, Race; Model 2: Adjusted for Age, Gender, Race, Education, Marital, PIR, Sedentary, Drinking status; Model 3: Adjusted for Age, Gender, Race, Education, Marital, PIR, Sedentary, Drinking status, Hyperlipidemia, CVD.

TyG: Triglyceride Glucose; TyG-BMI: Triglyceride Glucose - Body Mass Index; TyG-WHtR: Triglyceride Glucose - Waist to Height Ratio; TyG-WWI: Triglyceride Glucose - Weight Adjusted Waist Index; TyG-WC: Triglyceride Glucose - Waist Circumference; ABSI: A Body Shape Index; LAP: Lipid Accumulation Product; VAI: Visceral Adiposity Index.

# Table S3. Cardiovascular mortality HR based on quartiles of obesity and lipid-related indices

| **Cardiovascular mortality** | **Model 1**  **HR (95% CI)** | **Model 2**  **HR (95% CI)** | **Model3**  **HR (95% CI)** |
| --- | --- | --- | --- |
| **TyG** |  |  |  |
| Q1 (6.56,8.33) | Reference | Reference | Reference |
| Q2 (8.34,8.74) | 1.52(0.90, 2.56) | 1.37(0.79, 2.37) | 1.42(0.83, 2.44) |
| Q3 (8.75,9.17) | 0.93(0.55, 1.59) | 0.89(0.53, 1.51) | 0.89(0.50, 1.57) |
| Q4 (9.18,11.03) | 2.14(1.23, 3.71)** | 1.96(1.09, 3.53)* | 1.98(1.04, 3.76)* |
| **TyG(Per 1 unit increase)** | 1.57(1.09, 2.27)* | 1.53(1.04, 2.26)* | 1.57(1.04, 2.35)* |
| **TyG-BMI** |  |  |  |
| Q1 (115.40, 223.37) | Reference | Reference | Reference |
| Q2 (223.38, 262.31) | 0.67(0.41, 1.11) | 0.58(0.34, 1.00) | 0.55(0.32, 1.06) |
| Q3 (262.32, 313.23) | 0.89(0.51, 1.56) | 0.80(0.44, 1.48) | 0.73(0.39, 1.39) |
| Q4 (313.24, 620.83) | 1.18(0.73, 1.92) | 0.92(0.56, 1.52) | 0.82(0.47, 1.42) |
| **TyG-BMI(Per 1 unit increase)** | 1.00 (1.00, 1.00) | 1.00 (1.00, 1.00) | 1.00 (1.00, 1.00) |
| **TyG-WC** |  |  |  |
| Q1 (453.12, 792.26) | Reference | Reference | Reference |
| Q2 (792.27, 902.24) | 0.92(0.57, 1.50) | 0.84(0.50, 1.41) | 0.83(0.49, 1.38) |
| Q3 (902.25, 1025.15) | 0.9(0.59, 1.39) | 0.81(0.51, 1.28) | 0.76(0.47, 1.24) |
| Q4 (1025.16, 1648.81) | 1.52(0.96, 2.41) | 1.25(0.74, 2.11) | 1.15(0.65, 2.02) |
| **TyG-WC(Per 1 unit increase)** | 1.00 (1.00, 1.00) | 1.00 (1.00, 1.00) | 1.00 (1.00, 1.00) |
| **TyG-WHtR** |  |  |  |
| Q1 (2.63, 4.73) | Reference | Reference | Reference |
| Q2 (4.74, 5.40) | 0.90(0.52, 1.55) | 0.82(0.48, 1.40) | 0.78(0.45, 1.32) |
| Q3 (5.41, 6.14) | 1.17(0.63, 2.18) | 0.97(0.48, 1.96) | 0.88(0.42, 1.83) |
| Q4 (6.15, 10.23) | 1.41(0.85, 2.34) | 1.16(0.69, 1.94) | 1.03(0.59, 1.79) |
| TyG-WHtR | 1.22(0.99, 1.51) | 1.14(0.91, 1.43) | 1.10(0.86, 1.42) |
| **TyG-WWI** |  |  |  |
| Q1 (60.85, 90.83) | Reference | Reference | Reference |
| Q2 (90.84, 98.38) | 0.81(0.44, 1.50) | 0.69(0.37, 1.28) | 0.72(0.39, 1.32) |
| Q3 (98.39, 106.25) | 1.20(0.69, 2.09) | 1.33(0.69, 2.56) | 1.10(0.65, 1.86) |
| Q4 (106.26, 144.15) | 1.65(0.89, 3.03) | 1.33(0.69, 2.56) | 1.28(0.64, 2.52) |
| **TyG-WWI(Per 1 unit increase)** | 1.03(1.01, 1.04)** | 1.02(1.00, 1.04)* | 1.02(1.00, 1.04) |
| **ABSI** |  |  |  |
| Q1 (0.60, 0.78) | Reference | Reference | Reference |
| Q2 (0.79, 0.81) | 1.18(0.59, 2.38) | 1.13(0.56, 2.29) | 1.32(0.66, 2.63) |
| Q3 (0.82, 0.85) | 1.39(0.73, 2.65) | 1.44(0.75, 2.79) | 1.52(0.80, 2.92) |
| Q4 (0.86, 0.99) | 1.73(0.93, 3.23) | 1.66(0.87, 3.16) | 1.62(0.87, 3.01) |
| **ABSI(Per 1 unit increase)** | 32.5(1.32, 798)* | 22.2(0.80, 615) | 11.3(0.48, 270) |
| **LAP** |  |  |  |
| Q1 (3.07, 149.93) | Reference | Reference | Reference |
| Q2 (149.94, 202.77) | 0.56(0.35, 0.91)* | 0.56(0.34, 0.95)* | 0.60(0.36, 0.99)* |
| Q3 (202.78, 268.45) | 0.80(0.52, 1.24) | 0.75(0.48, 1.17) | 0.76(0.48, 1.20) |
| Q4 (268.46, 604.96) | 1.03(0.60, 1.76) | 0.90(0.49, 1.65) | 0.91(0.48, 1.73) |
| **LAP(Per 1 unit increase)** | 1.00 (1.00, 1.00) | 1.00 (1.00, 1.00) | 1.00 (1.00, 1.00) |
| **VAI** |  |  |  |
| Q1 (0.15, 1.01) | Reference | Reference | Reference |
| Q2 (1.02, 1.68) | 1.28(0.75, 2.17) | 1.21(0.69, 2.14) | 1.22(0.70, 2.15) |
| Q3 (1.69, 2.75) | 1.59(0.89, 2.84) | 1.51(0.80, 2.83) | 1.46(0.75, 2.82) |
| Q4 (2.76, 11.39) | 1.57(0.91, 2.73) | 1.51(0.86, 2.67) | 1.50(0.84, 2.68) |
| **VAI(Per 1 unit increase)** | 1.10 (0.97, 1.23) | 1.08(0.96, 1.21) | 1.07(0.96, 1.21) |

*P < 0.05; **P < 0.01; ***P < 0.001.

Multiple Cox regression model: Model 1: Adjusted for Age, Gender, Race; Model 2: Adjusted for Age, Gender, Race, Education, Marital, PIR, Sedentary, Drinking status; Model 3: Adjusted for Age, Gender, Race, Education, Marital, PIR, Sedentary, Drinking status, Hyperlipidemia, CVD.

TyG: Triglyceride Glucose; TyG-BMI: Triglyceride Glucose - Body Mass Index; TyG-WHtR: Triglyceride Glucose - Waist to Height Ratio; TyG-WWI: Triglyceride Glucose - Weight Adjusted Waist Index; TyG-WC: Triglyceride Glucose - Waist Circumference; ABSI: A Body Shape Index; LAP: Lipid Accumulation Product; VAI: Visceral Adiposity Index.

# Table S4. Cancer mortality HR based on quartiles of obesity and lipid-related indices

| **Cancer mortality** | **Model 1**  **HR (95% CI)** | **Model 2**  **HR (95% CI)** | **Model3**  **HR (95% CI)** |
| --- | --- | --- | --- |
| **TyG** |  |  |  |
| Q1 (6.56,8.33) | Reference | Reference | Reference |
| Q2 (8.34,8.74) | 0.99 (0.49,1.97) | 0.92 (0.45,1.86) | 0.96 (0.48,1.91) |
| Q3 (8.75,9.17) | 1.04 (0.51,2.14) | 0.93 (0.44,1.98) | 0.97 (0.47,2.01) |
| Q4 (9.18,11.03) | 1.49 (0.77,2.90) | 1.41 (0.67,2.97) | 1.49 (0.72,3.07) |
| **TyG(Per 1 unit increase)** | 1.34 (0.92,1.96) | 1.32 (0.86,2.02) | 1.36 (0.90,2.07) |
| **TyG-BMI** |  |  |  |
| Q1 (115.40, 223.37) | Reference | Reference | Reference |
| Q2 (223.38, 262.31) | 0.67 (0.37,1.24) | 0.76 (0.42,1.36) | 0.75 (0.42,1.36) |
| Q3 (262.32, 313.23) | 0.78 (0.44,1.40) | 1.07 (0.46,2.48) | 1.03 (0.44,2.45) |
| Q4 (313.24, 620.83) | 1.11 (0.62,1.99) | 1.86 (0.53,6.46) | 1.78 (0.51,6.19) |
| **TyG-BMI(Per 1 unit increase)** | 1.00 (1.00, 1.00) | 1.01 (1.00, 1.02) | 1.01 (1.00, 1.02) |
| **TyG-WC** |  |  |  |
| Q1 (453.12, 792.26) | Reference | Reference | Reference |
| Q2 (792.27, 902.24) | 1.05 (0.52,2.11) | 1.25 (0.62,2.54) | 1.29 (0.64,2.63) |
| Q3 (902.25, 1025.15) | 1.08 (0.56,2.07) | 1.59 (0.69,3.66) | 1.61 (0.71,3.64) |
| Q4 (1025.16, 1648.81) | 1.57 (0.83,2.96) | 3.04 (1.08,8.59)* | 3.09 (1.11,8.58)* |
| **TyG-WC(Per 1 unit increase)** | 1.00 (1.00,1.00) | 1.00 (1.00, 1.01) | 1.00 (1.00, 1.01)* |
| **TyG-WHtR** |  |  |  |
| Q1 (2.63, 4.73) | Reference | Reference | Reference |
| Q2 (4.74, 5.40) | 0.46 (0.23,0.92)* | 0.50 (0.25,1.00) | 0.51 (0.25,1.04) |
| Q3 (5.41, 6.14) | 1.00 (0.58,1.73) | 1.26 (0.59,2.67) | 1.27 (0.60,2.68) |
| Q4 (6.15, 10.23) | 0.99 (0.53,1.87) | 1.47 (0.47,4.64) | 1.46 (0.47,4.52) |
| TyG-WHtR | 1.07 (0.86,1.34) | 1.25 (0.81,1.92) | 1.25 (0.82,1.90) |
| **TyG-WWI** |  |  |  |
| Q1 (60.85, 90.83) | Reference | Reference | Reference |
| Q2 (90.84, 98.38) | 0.65 (0.30,1.42) | 0.60 (0.27,1.30) | 0.61 (0.27,1.40) |
| Q3 (98.39, 106.25) | 0.92 (0.45,1.86) | 0.86 (0.41,1.78) | 0.90 (0.43,1.89) |
| Q4 (106.26, 144.15) | 1.18 (0.58,2.41) | 1.17 (0.55,2.47) | 1.19 (0.56,2.55) |
| **TyG-WWI(Per 1 unit increase)** | 1.01 (0.99,1.03) | 1.01 (0.99,1.04) | 1.01 (0.99,1.04) |
| **ABSI** |  |  |  |
| Q1 (0.60, 0.78) | Reference | Reference | Reference |
| Q2 (0.79, 0.81) | 1.14 (0.42,3.15) | 1.14 (0.41,3.16) | 1.17 (0.42,3.25) |
| Q3 (0.82, 0.85) | 1.78 (0.75,4.20) | 1.85 (0.79,4.37) | 1.88 (0.79,4.48) |
| Q4 (0.86, 0.99) | 1.51 (0.65,3.48) | 1.52 (0.61,3.76) | 1.52 (0.62,3.74) |
| **ABSI(Per 1 unit increase)** | 106.19 (2.21,506.14) | 144.84 (23.33,1077.18) | 23.11 (12.33,111.05) |
| **LAP** |  |  |  |
| Q1 (3.07, 149.93) | Reference | Reference | Reference |
| Q2 (149.94, 202.77) | 0.56 (0.29,1.10) | 0.65 (0.33,1.31) | 0.67 (0.33,1.33) |
| Q3 (202.78, 268.45) | 0.88 (0.50,1.55) | 1.05 (0.56,1.96) | 1.07 (0.57,2.01) |
| Q4 (268.46, 604.96) | 0.89 (0.48,1.66) | 1.02 (0.52,1.99) | 1.05 (0.54,2.07) |
| **LAP(Per 1 unit increase)** | 1.00 (1.00, 1.00) | 1.00 (1.00, 1.00) | 1.00 (1.00, 1.00) |
| **VAI** |  |  |  |
| Q1 (0.15, 1.01) | Reference | Reference | Reference |
| Q2 (1.02, 1.68) | 0.90 (0.47,1.72) | 0.84 (0.42,1.66) | 0.86 (0.44,1.71) |
| Q3 (1.69, 2.75) | 1.17 (0.57,2.40) | 1.14 (0.54,2.40) | 1.21 (0.58,2.52) |
| Q4 (2.76, 11.39) | 1.68 (0.88,3.23) | 1.59 (0.78,3.27) | 1.74 (0.86,3.53) |
| **VAI(Per 1 unit increase)** | 1.15 (1.03,1.28)* | 1.14 (1.01,1.28)* | 1.15 (0.93,1.28) |

*P < 0.05; **P < 0.01; ***P < 0.001.

Multiple Cox regression model: Model 1: Adjusted for Age, Gender, Race; Model 2: Adjusted for Age, Gender, Race, Education, Marital, PIR, Sedentary, Drinking status; Model 3: Adjusted for Age, Gender, Race, Education, Marital, PIR, Sedentary, Drinking status, Hyperlipidemia, CVD.

TyG: Triglyceride Glucose; TyG-BMI: Triglyceride Glucose - Body Mass Index; TyG-WHtR: Triglyceride Glucose - Waist to Height Ratio; TyG-WWI: Triglyceride Glucose - Weight Adjusted Waist Index; TyG-WC: Triglyceride Glucose - Waist Circumference; ABSI: A Body Shape Index; LAP: Lipid Accumulation Product; VAI: Visceral Adiposity Index.

# Table S5. Indicators for Evaluating the Performance of Machine Learning Models

| Model | Accuracy | Precision | Recall | Roc_Auc |
| --- | --- | --- | --- | --- |
| logistic | 0.756157635 | 0.947513812 | 0.752192982 | 0.842225672 |
| enet | 0.756773399 | 0.947562098 | 0.752923977 | 0.844586646 |
| dt | 0.716133005 | 0.94157741 | 0.706871345 | 0.773438928 |
| rf | 0.772167488 | 0.938488576 | 0.780701754 | 0.837550827 |
| xgboost | 0.790024631 | 0.943333333 | 0.808479532 | 0.846285636 |
| rsvm | 0.764778325 | 0.9386121 | 0.77119883 | 0.831203171 |
| mlp | 0.744458128 | 0.946579194 | 0.738304094 | 0.846239949 |
| knn | 0.639162562 | 0.921336207 | 0.6254094 | 0.746186552 |

**Accuracy** = (TP + TN) / (TP + TN + FP + FN) = (TP + TN) / Count(Samples)

**Precision** = TP / (TP + FP), which is the ratio of true positives among all test data predicted as positive.

**Recall** = TP / (TP + FN)

**AUC** is used to quantify the area under the ROC curve, with values ranging from 0 to 1. The horizontal axis represents FPR, and the vertical axis represents TPR:

- True Positive Rate (TPR) = TP / (TP + FN)

- False Positive Rate (FPR) = FP / (FP + TN)

- TP: The number of samples that are actually Positive and predicted as Positive.

- FP: The number of samples that are actually Negative but predicted as Positive.

- TN: The number of samples that are actually Negative and predicted as Negative.

- FN: The number of samples that are actually Positive but predicted as Negative.

**DT**: Decision Tree; **Enet**: Elastic Net Regression; **KNN**: K-Nearest Neighbors; **Logistic**: Logistic Regression; **MLP**: Single Hidden Layer Neural Network; **RF**: Random Forest; **RSVM**: Robust Support Vector Machine; **XGBoost**: Extreme Gradient Boosting.

# Table S6. Characteristics of participants according to BMI.

| **Characteristic** | **Overall**, N = 4,058 (100%)^1^ | **Normal(≥18.5,<25)**  N = 799 (19%)^1,2^ | **Overweight(≥25,<30)**  N = 1,292 (31%)^1,2^ | **Obese(≥30)**  N = 1,935 (49%)^1,2^ | **P Value**^2^ |
| --- | --- | --- | --- | --- | --- |
| **BMI** | 31 (7) | 23 (2) | 27 (1) | 37 (6) | **<0.001** |
| **Age (years)** | 54.6 (16.5) | 56.6 (17.5) | 56.1 (16.4) | 52.8 (15.8) | **<0.001** |
| **Sex** |  |  |  |  | **0.009** |
| *Female* | 2,470 (58%) | 454 (58%) | 706 (52%) | 1,292 (62%) |  |
| *Male* | 1,588 (42%) | 345 (42%) | 586 (48%) | 643 (38%) |  |
| **Race** |  |  |  |  | **<0.001** |
| *Non-Hispanic White and Other* | 2,033 (72%) | 516 (80%) | 679 (74%) | 816 (68%) |  |
| *Hispanic* | 1,085 (15%) | 153 (12%) | 369 (16%) | 560 (16%) |  |
| *Non-Hispanic Black* | 940 (13%) | 130 (8.6%) | 244 (10%) | 559 (16%) |  |
| **Marital** |  |  |  |  | >0.9 |
| *Divorced* | 2,232 (59%) | 447 (57%) | 734 (59%) | 1,035 (59%) |  |
| *Married* | 1,677 (39%) | 325 (40%) | 510 (39%) | 828 (38%) |  |
| *Never married* | 149 (2.6%) | 27 (3.0%) | 48 (2.2%) | 72 (2.7%) |  |
| **Education** |  |  |  |  | **0.032** |
| *Below high school* | 599 (8.4%) | 99 (7.1%) | 217 (10%) | 280 (7.8%) |  |
| *High school graduate or GED* | 1,611 (40%) | 316 (40%) | 491 (38%) | 784 (41%) |  |
| *Some college or above* | 1,848 (51%) | 384 (53%) | 584 (52%) | 871 (51%) |  |
| **Drinking.status** |  |  |  |  | 0.2 |
| *Current drinker* | 1,538 (45%) | 280 (43%) | 476 (45%) | 768 (46%) |  |
| *Former drinker* | 1,095 (25%) | 203 (23%) | 338 (24%) | 546 (26%) |  |
| *Never-drinker* | 1,425 (30%) | 316 (34%) | 478 (30%) | 621 (28%) |  |
| **Hyperlipidemia** | 3,266 (80%) | 576 (72%) | 1,057 (79%) | 1,617 (84%) | **<0.001** |
| **Weight** | 86 (23) | 63 (9) | 77 (11) | 102 (20) | **<0.001** |
| **Height** | 166 (10) | 166 (10) | 167 (11) | 166 (10) | **0.039** |
| **Wistline** | 104 (17) | 85 (7) | 98 (7) | 116 (13) | **<0.001** |
| **Insulin** | 16 (18) | 8 (5) | 12 (12) | 22 (22) | **<0.001** |
| **Triglyceride** | 128 (67) | 106 (58) | 127 (67) | 138 (69) | **<0.001** |
| **Blood glucose** | 118 (38) | 113 (39) | 114 (34) | 122 (39) | **<0.001** |
| **TyG** | 8.76 (0.62) | 8.53 (0.62) | 8.73 (0.60) | 8.88 (0.60) | **<0.001** |
| **WHtR** | 0.63 (0.10) | 0.52 (0.04) | 0.58 (0.04) | 0.70 (0.08) | **<0.001** |
| **WWI** | 11.30 (0.80) | 10.85 (0.77) | 11.16 (0.75) | 11.59 (0.73) | **<0.001** |
| **TyG_BMI** | 273 (71) | 194 (21) | 240 (21) | 327 (60) | **<0.001** |
| **TyG_WHtR** | 5.51 (1.05) | 4.41 (0.56) | 5.11 (0.58) | 6.23 (0.87) | **<0.001** |
| **TyG_WWI** | 99 (11) | 93 (11) | 98 (10) | 103 (11) | **<0.001** |
| **TyG_WC** | 916 (174) | 729 (91) | 852 (93) | 1,035 (145) | **<0.001** |
| **TyG_WWI.quantile.var** |  |  |  |  | **<0.001** |
| *Q1* | 897 (25%) | 332 (48%) | 292 (29%) | 251 (13%) |  |
| *Q2* | 983 (25%) | 216 (27%) | 363 (27%) | 399 (23%) |  |
| *Q3* | 1,049 (25%) | 137 (14%) | 349 (26%) | 560 (29%) |  |
| *Q4* | 1,129 (25%) | 114 (11%) | 288 (18%) | 725 (35%) |  |
| **TyG_BMI.quantile.var** |  |  |  |  | **<0.001** |
| *Q1* | 1,000 (25%) | 708 (91%) | 257 (22%) | 3 (<0.1%) |  |
| *Q2* | 1,054 (25%) | 89 (8.4%) | 812 (62%) | 153 (8.1%) |  |
| *Q3* | 1,037 (25%) | 2 (0.2%) | 219 (16%) | 816 (41%) |  |
| *Q4* | 967 (25%) | 0 (0%) | 4 (0.2%) | 963 (51%) |  |
| **TyG_WHtR.quantile.var** |  |  |  |  | **<0.001** |
| *Q1* | 921 (25%) | 546 (74%) | 301 (28%) | 42 (2.4%) |  |
| *Q2* | 1,025 (25%) | 199 (21%) | 559 (44%) | 267 (15%) |  |
| *Q3* | 1,063 (25%) | 51 (4.1%) | 367 (24%) | 645 (34%) |  |
| *Q4* | 1,049 (25%) | 3 (<0.1%) | 65 (4.1%) | 981 (48%) |  |
| **TyG_WC.quantile.var** |  |  |  |  | **<0.001** |
| *Q1* | 992 (25%) | 581 (77%) | 317 (26%) | 63 (2.7%) |  |
| *Q2* | 1,102 (25%) | 181 (19%) | 606 (44%) | 314 (15%) |  |
| *Q3* | 1,018 (25%) | 35 (4.0%) | 326 (25%) | 657 (33%) |  |
| *Q4* | 946 (25%) | 2 (0.1%) | 43 (3.8%) | 901 (49%) |  |

1Mean ± SD for continuous; n (%) for categorical

2t-test adapted to complex survey samples; chi-squared test with Rao & Scott's second-order correction.

TyG: Triglyceride Glucose; TyG-BMI: Triglyceride Glucose - Body Mass Index; TyG-WHtR: Triglyceride Glucose - Waist to Height Ratio; TyG-WWI: Triglyceride Glucose - Weight Adjusted Waist Index; TyG-WC: Triglyceride Glucose - Waist Circumference; ABSI: A Body Shape Index; LAP: Lipid Accumulation Product; VAI: Visceral Adiposity Index.
